# Comprehensive identification of sphingolipid species by in silico retention time and tandem mass spectral library

**DOI:** 10.1186/s13321-017-0205-3

**Published:** 2017-03-15

**Authors:** Hiroshi Tsugawa, Kazutaka Ikeda, Wataru Tanaka, Yuya Senoo, Makoto Arita, Masanori Arita

**Affiliations:** 1 RIKEN Center for Sustainable Resource Science, 1-7-22 Suehiro-cho, Tsurumi-ku, Yokohama, Kanagawa 230-0045 Japan; 2 RIKEN Center for Integrative Medical Sciences, 1-7-22 Suehiro-cho, Tsurumi-ku, Yokohama, Kanagawa 230-0045 Japan; 3 Japan Agency for Medical Research and Development (AMED-PRIME), 1-7-1 Yomiuri Shimbun Building, Otemachi, Chiyoda-ku, Tokyo, 100-0004 Japan; 4 Graduate School of Medical Life Science, Yokohama City University, 1-7-29 Suehiro-cho, Tsurumi-ku, Yokohama, 230-0045 Japan; 5 Department of Genetics, SOKENDAI (The Graduate University for Advanced Studies), 1111 Yata, Mishima, Shizuoka 411-8540 Japan; 6 Division of Physiological Chemistry and Metabolism, Graduate School of Pharmaceutical Sciences, Keio University, 1-5-30 Shibakoen, Minato-ku, Tokyo, 105-8512 Japan; 7 National Institute of Genetics, 1111 Yata, Mishima, Shizuoka 411-8540 Japan

**Keywords:** In silico MS/MS, Retention time prediction, Mass fragmentation, Lipids

## Abstract

**Background:**

Liquid chromatography coupled with electrospray ionization tandem mass spectrometry (LC–ESI–MS/MS) is used for comprehensive metabolome and lipidome analyses. Compound identification relies on similarity matching of the retention time (RT), precursor *m/z*, isotopic ratio, and MS/MS spectrum with reference compounds. For sphingolipids, however, little information on the RT and MS/MS references is available.

**Results:**

Negative-ion ESI–MS/MS is a useful method for the structural characterization of sphingolipids. We created theoretical MS/MS spectra for 21 sphingolipid classes in human and mouse (109,448 molecules), with substructure-level annotation of unique fragment ions by MS-FINDER software. The existence of ceramides with β-hydroxy fatty acids was confirmed in mouse tissues based on cheminformatic- and quantum chemical evidences. The RT of sphingo- and glycerolipid species was also predicted for our LC condition. With this information, MS-DIAL software for untargeted metabolome profiling could identify 415 unique structures including 282 glycerolipids and 133 sphingolipids from human cells (HEK and HeLa) and mouse tissues (ear and liver).

**Conclusions:**

MS-DIAL and MS-FINDER software programs can identify 42 lipid classes (21 sphingo- and 21 glycerolipids) with the in silico RT and MS/MS library. The library is freely available as Microsoft Excel files at the software section of our RIKEN PRIMe website (http://prime.psc.riken.jp/).

**Electronic supplementary material:**

The online version of this article (doi:10.1186/s13321-017-0205-3) contains supplementary material, which is available to authorized users.

## Background

Liquid chromatography coupled with electrospray ionization tandem mass spectrometry (LC–ESI–MS/MS) is widely used for the comprehensive identification of small biomolecules [1]. Compound identification in untargeted metabolomics is based on similarity matching of four elements: the retention time (RT), precursor *m/z*, isotopic ratio, and MS/MS spectrum with reference compounds. Our MS-DIAL software integrates all four information types; it can process raw MS data of six major vendors (Agilent Technologies, Bruker Daltonics, Sciex, Shimadzu, Thermo Fisher Scientific, and Waters) for any data acquisition methods such as conventional LC/MS, data-dependent MS/MS, and data-independent MS/MS acquisitions [2]. It also exploits MS/MS databases such as MassBank (26,296 MS/MS spectra covering 3127 authentic compound structures) and NIST14 (234,284 MS/MS spectra covering 9344 authentic compound structures) for comprehensive metabolome analysis [3]. We can also predict the RT by the quantitative structure retention relationship (QSRR) in combination with multivariate analysis [4, 5], but these reference databases are not yet comprehensive: the human metabolome database (HMDB) contains 41,993 unique structures in contrast to their number in MassBank and NIST14 [6].

The structure elucidation of unknown MS/MS spectra via fragmentation models and rules is one way to improve such reference libraries [7–9]. An understanding of the fragmentation scheme in low-energy collision-induced dissociation (low-energy CID) facilitates the theoretic construction of the MS/MS spectra of small biomolecules [10]. A successful example is the LipidBlast library, a comprehensive construction of in silico MS/MS spectra for glycerolipids (and a few sphingolipids) [11]. The original LipidBlast library contained in silico MS/MS spectra for the triple quadrupole mass spectrometer (QqQMS) and the Fourier transform mass spectrometer (FT-ICR), covering 26 lipid classes with some adduct ion varieties. Since then the library has been expanded for quadrupole time-of-flight (QTOF) MS with classes such as 1,2-diacyl-3-*O*-α-glucuronosylglycerol (GlcADG) [12], fatty acid ester of hydroxyl fatty acids (FAHFA) [13], and diacylglyceryl-*N,N,N*-trimethylhomoserine (DGTS) [2]. The combination of LipidBlast and MS-DIAL software has identified 1023 glycerolipids in 9 algal species [2].

Here we introduce a theoretical MS/MS library of 21 sphingolipid classes for negative ionization mode-ESI–MS/MS [ESI(-)-MS/MS], 8 human ceramide classes [14], one murine ceramide class [15], their monoglycosides (HexCer), and sphingomyelin. For each class, the substructure of diagnostic fragment ions was identified with MS-FINDER software using hydrogen rearrangement (HR) rules (Table 1) [9] with detailed manual curation. Moreover, their RT was predicted for our LC condition that can effectively separate multiple lipid classes. We also show the comprehensive identification of glycero- and sphingolipids as the application of MS-DIAL software for 4 LC/MS/MS data: human cervical cancer (HeLa) cells, human embryonic kidney (HEK) cells, and liver- and ear tissues of C57BL/6 mice. Two libraries, one for glycerolipids and the other for sphingolipids, are stored as LipidBlast templates; they can be downloaded at the standalone software section of the RIKEN PRIMe website (http://prime.psc.riken.jp/).

**Table 1:** Summary of hydrogen rearrangement rules [9]

Rule ID	Ion mode	Equation^a,b,c^	Cleaved terminal element^d^
P1	Positive	M → [M′ − H]^+^	**C**, **P**, **S**
P2	Positive	M → [M′ + H]^+^	**N**, **O**, P, S
P3	Positive	M^+^ → M′^+^	C, **N**, **O**, P, S
P4	Positive	M^+^ → [M′ − 2H]^+^	**C**, N, O, **P**, **S**
N1	Negative	M → [M′ − H]^−^	C, **N**, **O**, P, S
N2	Negative	M → [M′ − 3H]^−^	**C**, **P**
N3	Negative	M → [M′ − 2H]^−^	**S**
N4	Negative	M^−^ → M′^−^	C, **N**, **O**, P, S
N5	Negative	M^−^ → [M′ − 2H]^−^	**C**, N, O, **P**, **S**

^a^M and M′ indicate the neutralized (hydrogen-supplemented) form

^b^Rules of P1, P2, N1, N2, and N3 consider the neutralized form as the precursor

^c^Rules of P3, P4, N4, and N5 consider the ionized form as the precursor

^d^Bold face indicates the most frequent pattern

The roles of our two programs, MS-DIAL and MS-FINDER, are as follows. MS-DIAL handles raw MS data for the ‘entire metabolome profiling’, and MS-FINDER uses MS/MS peaks in ASCII format, i.e. the pairs of *m/z* and intensity, for the structure elucidation from unknown spectra and for the substructure annotation of fragment ions. The spectral library in this work is used by both programs, which in combination allow us to automatically identify lipid molecules from the stage of raw MS data and from MS/MS peak lists.

## Results and discussion

### Building blocks of sphingolipids and their fragment prediction

**Fig. 1:**
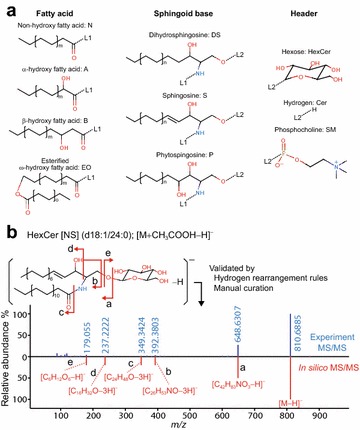
Building blocks of sphingolipid classes and an example of the in silico MS/MS spectrum. **a** The structural descriptions and abbreviations for fatty acids, sphingoid bases, and lipid classes are shown. The symbols *m*, *o*, and *n* describe the number of repeated substructures. The capitals *L1* and *L2* describe the connected modules among sphingoid bases (*L1* and *L2*), fatty acid (*L1*), and head moieties (*L2*). **b** The upper (*blue*) and lower (*red*) panels show the experimental MS/MS spectrum and the in silico MS/MS spectrum of HexCer [NS] (d18:1/24:0), respectively. The *red arrows* indicate bond cleavages. Associated formulas with rearranged hydrogens are shown for each labeled cleavage

The sphingolipid structure consists of 3 building blocks: fatty acid, the sphingoid base, and the head moiety (Fig. 1a). We focus on 4 types of fatty acid (non-hydroxy fatty acid [N], α-hydroxy fatty acid [A], β-hydroxy fatty acid [B], and esterified ω-hydroxy fatty acid [EO]), 3 sphingoid bases (dihydrosphingosine [DS], sphingosine [S], and phytosphingosine [P]), and 3 head moieties (hexose [HexCer], hydrogen [Cer], and phosphocholine [SM]) (Table 2). The symbols in brackets indicate the notation for each block used by Masukawa et al. [14]. We do not discuss 6-hydroxy sphingosine and gangliosides because their identification remains difficult in our analytic framework. The spectral annotations of the negative ion mode, ESI(-), in low-energy collision-induced dissociation (CID) were addressed because the characterization of sphingolipid classes is frequently performed by ESI(-) in the LC–ESI–MS/MS-based lipidomics approach.

**Table 2:** Summary of sphingolipid classes and MS/MS spectra developed in this study. Abbreviation source: Ref. [14]

Name	Abbreviation	Structure count	Spectra count	Adduct type
Sphingomyelin	SM	3384	6768	Formate, acetate
Ceramide non-hydroxyfatty acid-sphingosine	Cer [NS]	3360	10,080	Proton, formate, acetate
Glucosylceramide non-hydroxyfatty acid-sphingosine	HexCer [NS]	3360	10,080	Proton, formate, acetate
Ceramide non-hydroxyfatty acid-dihydrosphingosine	Cer [NDS]	1120	3360	Proton, formate, acetate
Glucosylceramide non-hydroxyfatty acid-dihydrosphingosine	HexCer [NDS]	1120	3360	Proton, formate, acetate
Ceramide α-hydroxy fatty acid-sphingosine	Cer [AS]	3360	10,080	Proton, formate, acetate
Glucosylceramide α-hydroxy fatty acid-sphingosine	HexCer [AS]	3360	10,080	Proton, formate, acetate
Ceramide α-hydroxy fatty acid-dihydrosphingosine	Cer [ADS]	1120	3360	Proton, formate, acetate
Glucosylceramide α-hydroxy fatty acid-dihydrosphingosine	HexCer [ADS]	1120	3360	Proton, formate, acetate
Ceramide β-hydroxy fatty acid-Sphingosine	Cer [BS]	3360	10,080	Proton, formate, acetate
Glucosylceramide β-hydroxy fatty acid-sphingosine	HexCer [BS]	3360	10,080	Proton, formate, acetate
Ceramide β-hydroxy fatty acid-dihydrosphingosine	Cer [BDS]	1120	3360	Proton, formate, acetate
Glucosylceramide β-hydroxy fatty acid-dihydrosphingosine	HexCer [BDS]	1120	3360	Proton, formate, acetate
Ceramide esterified ω-hydroxy fatty acid-sphingosine	Cer [EOS]	25,536	76,608	Proton, formate, acetate
Glucosylceramide esterified ω-hydroxy fatty acid-sphingosine	HexCer [EOS]	25,536	76,608	Proton, formate, acetate
Ceramide esterified ω-hydroxy fatty acid-dihydrosphingosine	Cer [EODS]	8512	25,536	Proton, formate, acetate
Glucosylceramide esterified ω-hydroxy fatty acid-dihydrosphingosine	HexCer [EODS]	8512	25,536	Proton, formate, acetate
Ceramide a-hydroxy fatty acid-phytospingosine	Cer [AP]	2772	8316	Proton, formate, acetate
Glucosylceramide a-hydroxy fatty acid-phytospingosine	HexCer [AP]	2772	8316	Proton, formate, acetate
Ceramide non-hydroxyfatty acid-phytospingosine	Cer [NP]	2772	8316	Proton, formate, acetate
Glucosylceramide non-hydroxyfatty acid-phytospingosine	HexCer [NP]	2772	8316	Proton, formate, acetate

Theoretical fragmentation for each sphingolipid class was extrapolated from the pattern of reference compounds. Structural analyses to determine the acyl chain length, double-bond count, and lipid class can be performed by the interpretation of the MS/MS spectrum. The mass fragmentation per lipid class is highly conserved among their acyl chain varieties. Figure 1b shows an example for HexCer [NS] (d18:1/24:0) of the acetate adduct. The bond cleavage position and the HR in the cleavage were determined with the computational MS/MS fragmentation program MS-FINDER, which implements the HR rules [9]. The results of fragment assignment were manually checked for multiple acyl chain varieties in the same sphingolipid class and reliably detected fragment ions were registered as diagnostic ions. Each fragment ion was annotated as a neutralized (i.e., valence-satisfied) structure plus or minus hydrogen(s) and shown as [M ± aH]^+^ or [M ± bH]^−^, where M stands for the neutralized structure, and a/b for the number of rearranged hydrogens. Ion abundances were determined by the heuristic model [11] from experimental data obtained in this study **(**see “Methods/experimental”).

### Fragmentation of sphingolipids in negative ion mode

Here we introduce the substructure assignments of two ceramide species, Ceramide [NS] (d18:1/26:0) and Ceramide [AS] (d18:1/16:0). Other species were determined similarly; their details are presented in Additional file 1: Fig. S1.

**Fig. 2:**
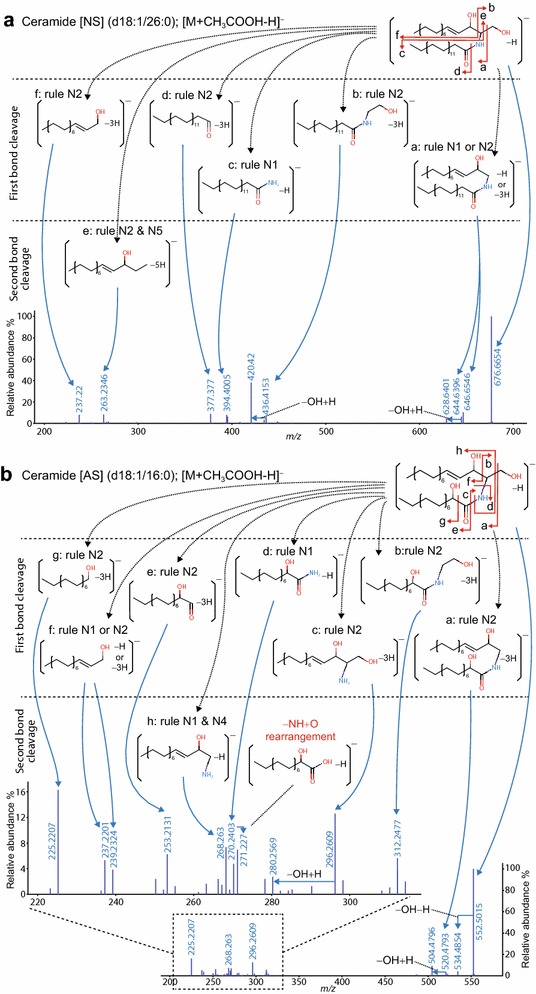
MS/MS annotations of two ceramides in negative ion mode of low energy CID: **a** ceramide [NS] (d18:1/26:0) and **b** ceramide [AS] (d18:1/16:0) as acetate adduct forms. The substructure ions were combinatorially assigned with hydrogen rearrangement (HR) rules (Table 1). Structures were depicted as the neutralized form, and the total count of rearranged hydrogens was assigned. The fragment ion arising from the internal rearrangement, which cannot yet be assigned by HR rules, is depicted in *red*

Fragment annotation of ceramide [NS], the major ceramide class, was performed with the MS-FINDER program in combination with substantial manual curation (Fig. 2a). The bond cleavage and the counts of hydrogen rearrangement were determined by the HR rules (Table 1). A total of 10 diagnostic ions was considered to be reliable fragment ions to define the Ceramide [NS] class: three (**b**, **c,** and **d**) and two (**e** and **f**) fragment ions in addition to their dehydration ions were the specific (unique) ions of fatty acid and of the sphingoid base, respectively. The assignments of fragments **a**, **b**, **d**, and **f** were annotated as a bond cleavage and the formation of a new double bond (one proton loss plus two hydrogen losses), formulated as HR rules N2. Fragments **a** and **c** were explained as the result of one proton loss (HR rule N1), and fragment **e** was assigned as the result of two bond cleavages and the formation of two new double bonds (or rings), formulated as the combination of HR rule N2 and N5 (one proton loss plus four hydrogen losses).

We also demonstrate the fragmentation of Ceramide with α-hydroxy fatty acid, Ceramide [AS] (d18:1/16:0), to describe the specificity of mass fragmentation compared to the Ceramide [NS] MS/MS spectra. We assigned a total of 14 diagnostic ions as the reliable and common ions of Ceramide [AS]. Most fragment ions (**a**, **b**, **c**, **e**, **f**, **g**) were generated by a bond cleavage and the formation of a new double bond (one proton loss plus two hydrogen losses), formulated as HR rule N2. Fragments **d** and **f** were explained as the result of one proton loss (HR rule N1), and fragment **h** was assigned as the result of two bond cleavages of one proton loss (the combination of HR rules N1 and N3). A unique fragment, *m/z* 271.227, was frequently monitored in the fragmentation of ceramides in ESI(-)-MS/MS [16] and considered to be the result of a nucleophilic substitution reaction: a nucleophilic hydroxyl anion of sphingoid moiety reacts with the ketone carbon of the fatty acid moiety. This fragment ion can also be monitored in Ceramide [NS] although the ion abundance is too low to be detected.

### RT prediction for the highly ‘step-formed’ chromatographic condition

The RT is essential for filtering out false-positive metabolites and for distinguishing the isomers of target molecules. The quantitative structure retention-relationship (QSRR) approach is one of the ‘golden’ techniques for predicting the RT of small biomolecules.

The lipid metabolites of murine ear tissue were extracted and analyzed with our LC/MS/MS technique in both positive- and negative ion mode **(**see “Methods/experimental”). Identification was performed with MS-DIAL version 2.24. A total of 284 identified lipids, including 12 sphingolipid- and 13 glycerolipid classes, was used as the training set (Additional file 2: Table S1).

**Fig. 3:**
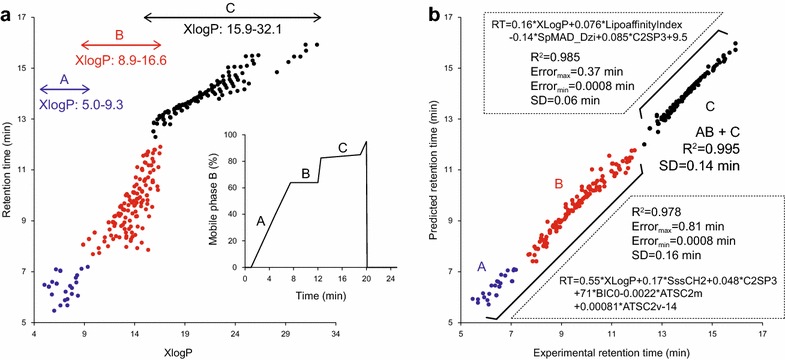
Retention time prediction of lipids in reverse-phase liquid chromatography. **a** The *x*- and *y*-axes show the calculated LogP (XLogP) and the experimental retention time of lipid molecules. The LC gradient condition is shown in the *right panel*. The eluted lipids in gradient stage A, B, and C are depicted as *blue*, *red*, and *black circles*, respectively. **b** The *x*- and *y*-axes show the experimental- and predicted retention times, respectively. The *colors* indicate the gradient stage. Two equations for retention time predictions of stage A + B and stage C are shown. *SD* is the standard deviation of the prediction errors

Using the PaDEL program [17], 2325 chemical descriptors were calculated on the basis of two dimensional structural information. We first examined the relationship between the calculated *Log P* (octanol–water partitioning coefficient) and the RT of identified lipids because the *Log P* value is known to correlate with RT in reverse-phase LC [18] (Fig. 3a). We used XLogP as its estimation [19]. Our findings suggested that 1) XlogP alone is not enough for the RT prediction, and 2) the elution profiles are substantially different between the LC gradients, stage B (isocratic condition) and stage C (gentle gradient condition) (see Fig. 3a, right panel).

To construct regression models separately for our step-forming chromatographic condition, we separated the 284 lipids into 168 and 116 lipid sets for range A + B (XLogP: 5.0–16.6) and range C (XLogP: 15.9–32.1), respectively. We first selected 47 descriptors on the basis of the correlation coefficient between the RT and each descriptor (threshold ≥ 0.85), and applied a multiple regression model with the ‘forward-step’ function for each set (see “Methods/experimental”). Six and four descriptors were selected for the ranges A + B and C, respectively (Fig. 3b). In the prediction model of the range A + B, an electrotopological state index (SssCH2) [20], a carbon type descriptor (C2SP3) [17], an information content descriptor (BIC0) [21], and two autocorrelation descriptors (ATSC2 m and ATSC2v) [21] were utilized in addition to a hydrophobicity value XLogP. The first three descriptors SssCH2, C2SP3, and BIC0 can be interpreted as the effects of carbon connectivity, considering the electronic state in the -CH2- moiety, the *sp3* hybrid orbital, and the complexity of bond graphs, respectively. Two autocorrelation descriptors, ATSC2 m and ATSC2v, describe the repeated substructure in a molecule, providing information on the acyl chain properties. On the other hand, a different descriptor for hydrophobicity was used to model range C (Lipoaffinity index) [22]. A Barysz distance matrix-based descriptor (SpMAD_Dzi) describing the ionization potential of heteroatoms was also used [21]. Consequently, the integration of hydrophobicity (XLogP and LipoaffinityIndex), the environment of the carbon atom (C2SP3, SssCH2, BIC0), and the electronic state of the heteroatom (SpMAD_Dzi) was important for the RT prediction of lipids in our reverse phase LC. The predicted RT exhibited a standard deviation (SD) of 0.14 min, an acceptable error for the filtering of compound identifications.

### Annotation of β-hydroxy ceramide, ceramide [BS], with chemoinformatic and quantum chemical evidence

**Fig. 4:**
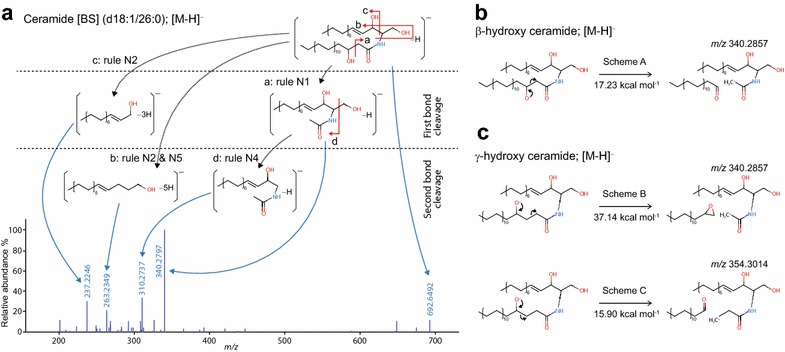
Structure elucidation of β-hydroxy fatty acid ceramide. **a** The experimental MS/MS spectrum, which was annotated as Ceramide [BS] (d18:1/26:0) by mass spectrometry experts, is shown in the *bottom panel*. The substructure ions with rearranged hydrogens were assigned to the diagnostic ions for ceramide [BS]. **b** The brief scheme to generate the base peak ion, *m/z* 340.2797 in (**a**), from the ceramide [BS] structure is shown as scheme A with the heat of formation calculated by the MOPAC program. **c** The brief scheme to generate the base peak from γ-hydroxy fatty acid ceramide is shown as scheme B with the heat of formation. In addition, the practical scheme arising from the α-elimination process at the hydroxy moiety is described as scheme C. These heat of formation data were used as important evidence to define the unknown lipid structure as ceramide [BS] from the mass fragmentation patterns

In Fig. 4, we emphasize the utility of the in silico RT and the MS/MS approach for Ceramide [BS] (d18:1/26:0). While there is no authentic standard for some lipid classes including a ceramide with β-hydroxy fatty acid, we concluded the existence of Ceramide [BS] species in murine ear tissue for the following reasons [15]. First, the RT of ceramide [BS] is earlier in reverse-phase LC than that of ceramide [AS] of the same precursor mass. The *Log P* value is known to correlate with RT in reverse phase LC [18], and the calculated Log *P* value (XLogP [19]) was smaller for β-hydroxy fatty acid (10.216 at Ceramide [BS] (d16:1/14:0)) than for α-hydroxy fatty acid {10.693 at Ceramide [AS] (d16:1/14:0)}. Second, in the ESI(+)-MS/MS spectrum, we detected up to two dehydrations (–2H_2_O) from the same ceramide whose spectrum is clearly different from that of phytosphingosine (Additional file 1: Fig. S1). In other words, the unique sphingosine fragmentat ions were monitored in ESI-(+)-MS/MS (Additional file 3: Fig. S2). For validation, we examined the fragmentation scheme to generate the base peak ion *m/z* 340.2797 in Fig. 4a using a quantum chemistry approach. Since we could not find any unique fragment in the MS/MS spectrum to define the hydroxylated position, we posited the possibility of two structures, β- or γ-hydroxy fatty acid ceramides, as two candidates. In fact, *m/z* 340.2797 can be explained as the result of a simple α-elimination process where the reaction is known in negative ion mode of low-energy CID. The α-elimination process to generate the base peak ion of β-hydroxy ceramide (Scheme A in Fig. 4b) was compared with a fragmentation process of γ-hydroxy ceramide to generate the same ion (Scheme B in Fig. 4c). An α-elimination process in γ-hydroxy ceramide (Scheme C in Fig. 4c) that results in a fragment different from *m/z* 340.2797 was also considered. Changes in the heat of formation along with the fragmentation processes were calculated and energetic barriers were evaluated using the semi-empirical PM7 method implemented in MOPAC2016 [23]. Scheme B required higher energy than scheme A (37.14 vs 17.23 kcal mol^−1^). In contrast, scheme C required almost the same amount of energy (15.90 kcal mol^−1^) as scheme A. Since γ-hydroxy ceramide prefers scheme C (α-elimination), the fragment ion of *m/z* 340.2797 cannot be produced from γ-hydroxy ceramide through scheme B. In addition, as β- and γ-hydroxy ceramide preferred the α-elimination process, it is likely that ceramide hydroxylated on other positions on its fatty acid moiety also prefers that process. Thus, the base peak ion can be regarded as an ion specific for β-hydroxy ceramide.

### Application of the in silico RT and MS/MS library to biological samples

The in silico RT and MS/MS spectra developed in this study were used in both MS-DIAL and MS-FINDER to compute spectral similarity as the dot product. The library currently contains the unique lipid structures of 42 lipid classes (21 glycerolipids and 21 sphingolipids) that can deal with both positive and negative ion modes, and several adduct types such as proton (H^+/−^), ammonium (NH_4_^+^), sodium (Na^+^), formate (HCOO^−^), and acetate (CH_3_COO^−^) adducts. The peak intensity of diagnostic ions (normalized to 0–999) was empirically determined by checking representative MS/MS spectra in each lipid class, and the intensity was shared among all lipids in the same class. The library can be downloaded as LipidBlast templates at the standalone software section of the RIKEN PRIMe website (http://prime.psc.riken.jp/).

**Fig. 5:**
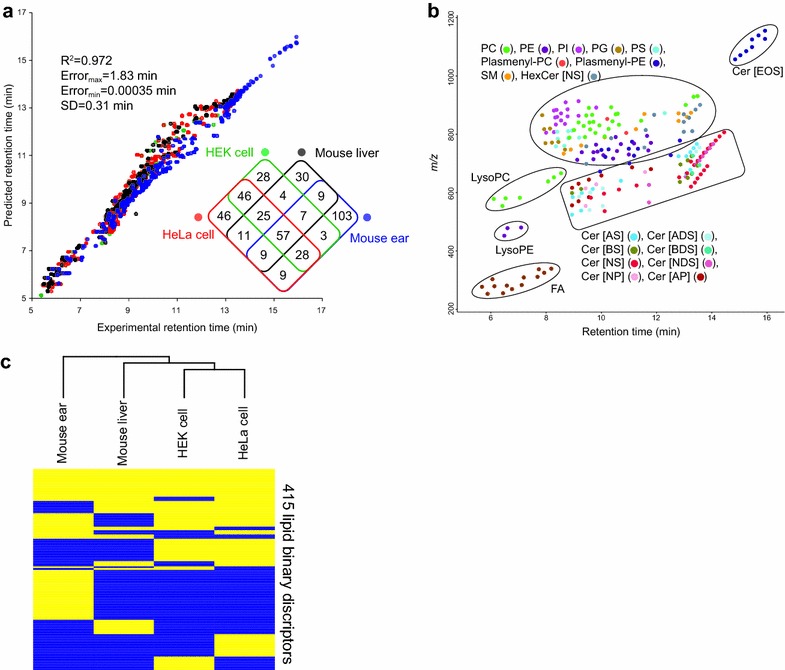
Lipid identification in four biological samples. **a** The *x*- and *y*-axes show the experimental- and predicted retention times of newly identified lipid molecules in four biological samples. The lipid molecules from HEK cells, HeLa cells, mouse liver tissue, and mouse ear tissue are depicted in *green*, *red*, *black*, and *blue*, respectively. The Venn diagram to describe the overlap of unique lipid structures is shown in the *right panel*. **b** Result of lipid profiling in a mouse ear tissue. The *x*- and *y*-axes show the retention time and *m/z*, respectively. Each spot indicates the lipid molecules detected by the MS-DIAL program, the *colors* identify each lipid class. **c** Result of hierarchical clustering analysis for four biological samples and 415 binary variables of lipids. *Yellow* and *blue* indicate ‘detected’ and ‘not detected’ for each sample, respectively. The species between human cells and mouse tissues are clearly separated by the lipid properties

To avoid overfitting to particular analytical batches, biological tissues, or column lot differences, four additional LC-ESI(-)-MS/MS data sets were evaluated: murine ear tissue (two replicates, one of which was used as the training set for RT prediction; analyzed on February 6, 2015), murine liver tissue (six replicates analyzed on June 15, 2016), HEK cells (three replicates analyzed on December 9, 2015), and HeLa cells (three replicates analyzed on December 9, 2015). All results were manually checked and total 415 unique lipid structures were confirmed (Table 3; Additional file 2: Tables S2, S3, S4, and S5). For these 415 structures, the average rate of false discovery (FP/(TP + FP)) and true positive (TP/(TP + FN)) was 5.6 and 99.1%, respectively. The standard deviation of predicted RT errors was 0.31 min although the LC columns of different lot numbers were used for the analyses of (1) ear tissue, (2) HEK and HeLa cells, and (3) liver tissue (Fig. 5a). The accuracy of RT predictions was not high enough to distinguish the structural isomers such as cis/trans and acyl chain positions because the RT differences of their isomers are practically less than 10 s. However, these errors are attributable to the irreproducibility of LC column lots because we encountered large differences in the experimental RT of several lipid molecules among our analyses (maximal 1.1 min RT difference, Additional file 2: Tables S2, S3, S4, S5).

**Table 3:** Summary of identified lipids in four biological samples

Class	HEK	HeLa	Mouse liver	Mouse ear
Cer [ADS]	0	0	0	6
Cer [AS]	0	0	0	7
Cer [BDS]	0	0	0	4
Cer [BS]	0	0	0	6
Cer [EOS]	0	0	0	12
Cer [NDS]	0	2	1	11
Cer [NS]	17	27	14	19
Cer [NP]	1	2	1	3
Cer [AP]	0	0	1	11
HexCer [NDS]	0	0	0	4
HexCer [NS]	7	6	5	12
SM	6	10	6	3
LysoPC	5	10	6	7
LysoPE	3	4	5	3
PC	38	29	35	32
PE	30	29	31	23
PG	12	14	10	7
PI	29	32	18	16
PS	8	17	1	10
Plasmenyl-PC	6	5	0	3
Plasmenyl-PE	19	20	6	12
FA	17	24	12	14
Total	198	231	152	225

In fact, the predicted RTs were powerful for filtering out many false-positives, and the combination of the in silico RT and MS/MS spectrum facilitated the determination of the lipid class, acyl chain lengths, and double-bond counts. In the lipidome data of murine ear tissue analyzed by LC-ESI(-)-MS/MS, 21 lipid classes, including 10 glycerolipid- and 11 sphingolipids, were simultaneously identified in a single run (Fig. 5b; Additional file 4: Fig. S3). Although there was a drastic change in the RT behavior of ceramide species at around 12 min on the chromatogram, MS-DIAL could correctly assign these lipids based on predicted RT information and theoretical MS/MS spectra.

Lastly, we discuss the lipid distribution in four biological samples (Fig. 5c; Additional file 5: Fig. S4). Note that there are false-negatives due to 1) MS sensitivities and 2) incompleteness of MS/MS spectra in the data-dependent acquisition mode: the only lipids we could identify by the checking the RT and MS/MS spectrum are shown. For example, determination of acyl moieties in the sphingomyelin (SM) class is difficult because the ion abundance of sphingoid base fragment ions is rarely monitored in our MS/MS setting although we can identify the lipid class as SM by the class specific diagnostic ions. Nevertheless, hierarchical clustering analysis (HCA) on the basis of a binary vector matrix of lipids (lipid descriptors) [2] reflected differences in their biological background (Fig. 5c). The glycerolipids containing even-length fatty acid chains from 16 to 24 and sphingolipids containing sphingosine d18:1 were the common chains among the four biological samples. Sphingadienine (d18:2) containing Ceramide [NS] was frequently monitored in HEK- and HeLa cells. The diversity of acyl chains, especially in phosphatidylinositol (PI), phosphatidylserine (PS), and Ceramide [NS] was larger in HeLa cells than the other samples. This can be interpreted as the overexpression of gene functions in cancer cells. On the other hand, many odd-length lipid chains (mostly 17:0 and 17:1) were identified in murine liver and ear tissues; they can be catalyzed from the diet and synthesized by microbiota. Likewise, the variety of ceramide classes was larger in murine ear tissues than in the other samples: sphingolipids including Cer [AS], Cer [ADS], Cer [BS], Cer [BDS], Cer [EOS], and HexCer [NDS] were identified in murine ear tissue. This observation suggests that the barrier functions in skin are regulated by a combination of ceramide classes and their acyl chain moieties.

## Conclusions

The use of the enriched in silico RT and MS/MS database was demonstrated as the application of MS-DIAL software. Currently, 42 lipid classes, including 21 glycerolipids and 21 sphingolipids, can be identified in MS-DIAL. Their diagnostic ions were theoretically assigned by hydrogen rearrangement rules (implemented in MS-FINDER) in combination with manual curation (Additional file 1: Fig. S1, Additional file 3: Fig. S2, Additional file 4: Fig. S3). Although their ion abundances were empirically optimized in the Sciex QTOFMS instrument at the collision energy of 40 with a 15 spread, the MS/MS library has been successfully utilized on other machines such as Waters-, Brucker-, and Agilent QTOF-MS, and on Thermo Q-Exactive with the optimization of their MS conditions (data not shown).

Some issues need improvement for the MS/MS-oriented identification of lipid species. To deal with all lipid diversities, we do not require all structures in the MS/MS database, i.e. all combinations of fatty acids, sphingoid bases, and lipid classes. Instead, the dictionary of fatty acid- and sphingoid chains and lipid classes may be sufficient for the generation and consideration of all lipid molecules. This would substantially reduce the database size, thereby reducing the random access memory (RAM) space. Furthermore, rule-based identification can be implemented to determine the identification level of the target lipid molecules like 1) PC (16:0/18:1(9Z)), if the MS/MS spectrum and retention time are confirmed by an authentic standard, 2) PC (16:0/18:1), if all diagnostic fragments for a lipid class and acyl chain are monitored, and 3) PC 34:1, if only diagnostic fragments to determine the lipid class are monitored. These computational improvements in MS-DIAL will be addressed elsewhere. Finally, we highly recommend sharing the in silico MS/MS of novel lipid classes by the metabolomics community for the comprehensive identification of small biomolecules.

## Methods/experimental

### Structure generation for sphingolipid molecules

All ceramide structures were generated by the SMILES codes for fatty acid, sphingoid, and head moieties. The acyl chain lengths of fatty acid and sphingoid moieties ranged 12–36 and 14–30, respectively. The double-bond counts of fatty acid and sphingoid moieties ranged 0–3 and 0–7, respectively. Among structural isomers, one SMILES code was chosen as the ‘representative’ structure. The double bond of fatty acid was regarded as *cis* conformation while the double bond of sphingoid was regarded as *trans*. The bond positions were determined by the information from literature and LipidMaps (http://www.lipidmaps.org). The SMILES codes were used to calculate their retention times. Integration was performed with a Microsoft Excel macro. The SMILES file was converted to an SDF file, and the SDF file to a SMILES file for formatting as daylight SMILES by means of ChemAxon Molconvert. The structures of sphingomyelin were downloaded from the LipidMaps website (http://www.lipidmaps.org/). The exact mass, formula, and InChIKey were generated by the ChemAxon Calculator. The precursor *m/z* of each lipid molecule was calculated as the proton adduct/loss, formate adduct, and acetate adduct by Microsoft Excel.

### MS/MS databases and fragment annotations

Over 1000 experimental measurements including reference compounds were performed by our LC/MS/MS condition. MS-FINDER version 1.76 was used to assign the substructure ions to the experimental fragment ions. Mass tolerance was set to 10 mDa, and the tree depth was set to two for in silico fragmentations. To reduce false-positive annotations, several lipid molecules containing different numbers of carbon atoms and double bonds were manually checked for each lipid class and the common features were extracted. The *m/z* values of the substructure ions were calculated in Microsoft Excel; they were managed as modified LipidBlast templates. The ion abundances were heuristically optimized by our LC/MS/MS condition as described elsewhere [11]: the collision energy and collision energy spread were set to 40 and 15 in the Sciex TripleTOF 5600 system, respectively.

### RT prediction for lipids

Two modified LipidBlast templates, one for glycerolipids and the other for sphingolipids, are managed in our lipidomics project (http://prime.psc.riken.jp). All of the SMILES code included in the templates was converted to SDF files. The PaDEL descriptor software was utilized to calculate one- and two-dimensional molecular descriptors and PubChem fingerprints from the SDF files [17]. Redundant and uniform variables were excluded, and the correlation coefficients between the RT and descriptors were calculated. Finally, 47 descriptors whose correlation coefficient exceeded 0.85 were used as predictor variables in regression analysis. The RT information of 284 lipids was used for model development. These lipid sets were separated based on the LC gradient stage (right panel in Fig. 3a) into 168- and 116 lipid sets for the range A + B (XLogP: 5.0–16.6) and C (XLogP: 15.9–32.1), respectively. A multiple regression model was used for RT predictions. The ‘forward-step’ function which applies the Akaike Information Criterion (AIC) for model selection was used to determine important variables. Six (XLogP, SssCH2, C2SP3, BIC0, ATSC2 m, ATSC2v) and four (XLogP, LipoaffinityIndex, SpMAD_Dzi, C2SP3) descriptors were used for range A + B and range C, respectively (Fig. 3b). The coefficients of XLogP, SssCH2, C2SP3, BIC0, ATSC2 m, ATSC2v, and the intercept in stage A + B were 0.55, 0.17, 0.048, 71, −0.0022, 0.00081, −14, respectively. The coefficients of XLogP, LipoaffinityIndex, SpMAD_Dzi, C2SP3, and the intercept in stage C were 0.16, 0.076, −0.14, 0.085, and 9.5, respectively. The RT calculation of lipid molecules included in the modified LipidBlast templates was based on the XLogP value: the equation of range A + B was applied at XLogP <= 15.9, the equation of range C at XLogP > = 16.6, and the average of two equations was applied at 15.9 < XLogP < 16.6. RT information of 1946 newly identified lipids from four biological samples was used for validating that accurate precursor ion masses and MS/MS spectra were confirmed by RT matching.

### MS-DIAL software and data processing parameters

The program of MS-DIAL version 2.24 was used in this study. The in silico RT and MS/MS spectra of 42 lipid classes (21 glycerolipids and 21 sphingolipids from LipidBlast templates) were implemented in MS-DIAL and MS-FINDER. The chain length for fatty acid and sphingoid was restricted to10–26 and 16–22, respectively, to be used for mammalian cells. The raw MS files (WIFF format file) were converted to ABF (analysis base file format) using the freely available Reifycs file converter (http://www.reifycs.com/AbfConverter/). The ABF files were imported into MS-DIAL, and the parameters were set as follows: (data collection) RT begin, 0 min; retention time end, 17 min; mass range begin, 0 Da; mass range end, 1500 Da; MS1 tolerance, 0.01 Da; MS2 tolerance, 0.05 Da; (peak detection) smoothing method, linear weighted moving average; smoothing level, 2 scan; minimum peak width, 5 scan; minimum peak height, 1000 amplitude; mass slice width, 0.1 Da; exclusion mass list, none; (identification) retention time tolerance, 2 min; MS1 accurate mass tolerance, 0.01 Da; MS2 accurate mass tolerance, 0.05 Da; identification score cut off, 80%.

### Compound identification

All diagnostic ions that determine the lipid class, acyl chain length, and double-bond count were manually checked in combination with the predicted RT information; 432, 560, 535, and 419 lipids were annotated in HEK cells (three replicates), HeLa cells (three replicates), murine liver tissues (six replicates), and murine ear tissues (two replicates), respectively (Additional file 2: Tables S2, S3, S4, and S5). The false discovery rate (FDR) and true positive rate (TPR) of the automated MS-DIAL output were calculated for 415 reliably identified lipid molecules. The total 1946 lipids were integrated by disregarding the acyl chain positions (*sn1, sn2, sn3*), double bond positions, and stereoisomers (*E, Z*) as described elsewhere [2]. For the remaining 415 lipids, the presence or absence in each of four biological samples was represented as a 415 × 4 binary data matrix (Additional file 2: Table S6). Hierarchical clustering analysis was performed using R statistical language (http://www.R-project.org) and the package ‘amap’ (http://CRAN.R-project.org/package=amap). The distance was calculated by ‘binary’ in the package and linkage was performed by ‘single’.

### Reagent and sample preparation

The preparation and extraction procedures of the biological samples were as described by Yokomizo et al. [24]. Methanol (MeOH), isopropanol (IPA), and acetonitrile (Ace) of LC–MS grade were purchased from FLUKA, and ammonium acetate and EDTA from Wako and Dojindo, respectively. Milli-Q water was purchased from Millipore. The bead pulverizing machine and 2-mL glass tubes were purchased from Bertin Technologies and FCR&Bio, respectively. Authentic standard compounds, Ceramide [NS] (d18:1/25:0), [AS] (d18:1/24:1), [AP] (t18:0/24:0), and [NP] (d18:1/24:0), glucosyl Ceramide [NS] (d18:1/24:1), and SM (d18:1/12:0) were purchased from Avanti Polar Lipids Inc. HEK293- and HeLa cell lines were cultured in D-MEM (high-glucose) with l-glutamine (Wako) containing 10% fetal bovine serum (FBS) and Pen Strep glutamine (PSG), and incubated in 5% CO_2_ at 37 °C in six-well cell culture multiwell plates (Greiner bio-one). After 1 h-cultivation, the medium was aspirated, cells were washed on the plates with 10 mM Tris–HCl (pH 8.0) buffered saline. The solution was transferred to a new glass tube, and then dried with a vacuum dryer. Liver and ear tissues of C57BL/6 J mice (CLEA Japan, Tokyo, Japan) were harvested according to the ethical protocol approved by the RIKEN Center for Integrative Medical Sciences. Tissues were frozen immediately after dissection and stored at −80 °C until lipid extraction.

For lipid extraction of HEK- and HeLa cells, chloroform (100 μL) was added to dried cells in a tube, followed by 30-s sonication. After 60-min incubation at room temperature, 200 μL of MeOH were added and vortexed for 10 s. After 120-min incubation we added 20 μL of Milli-Q water, vortexed again, and left the tube to stand for 10 min. The cells were then centrifuged at 2000×*g* for 10 min at 20 °C. Supernatant was transferred to LC/MS vials to determine the phosphorous contents of the lipid fraction. The phosphorus content of the extracted lipids was quantified by the method of Bartlett [25], and then the extracted lipids from HEK and HeLa cells were gently dried with N_2_ and reconstituted to 800 μM and 1 mM phosphorus with chloroform (methanol = 1:2), and stored at −80 °C until use. For lipid extraction of liver tissue, 144.8 mg in methanol (50 mg tissue/mL) were pulverized and homogenized in a bead pulverizing machine (6000 rpm for 15 s, ×2). Then 1200 μL of homogenized solvent were transferred to a 2-ml glass tube, 600 μL of chloroform were added and the tubes were vortexed for 10 s. After 60-min incubation at room temperature, 120 μL of Milli-Q water were added, and the tubes were again vortexed for 10 s, incubated for 15 min at room temperature, and centrifuged at 2000*g* for 10 min at 20 °C. The supernatant was transferred to LC/MS vials. For lipid extraction of ear tissue, 29.8 mg in methanol (29.8 mg tissue/mL) were pulverized and homogenized in a bead pulverizing machine (6500 rpm for 15 s, ×2), 200 μL of homogenized solvent were transferred to a 2-ml glass tube, 100 μL of chloroform were added, and the tubes were vortexed for 10 s. After 60-min incubation at room temperature, 20 μL of Milli-Q water were added, the tubes were vortexed for 10 s, incubated for 15 min at room temperature, and centrifuged at 2000*g* for 10 min at 20 °C. The supernatant was transferred to LC/MS vials.

### Analytical conditions

The liquid chromatography system consisted of a Waters Acquity UPLC system (Waters Inc.). Mobile phase A was 1:1:3 acetonitrile:methanol:water (v/v/v) with 5 mM ammonium acetate and 10 nM EDTA. Mobile phase B was 100% isopropanol with 5 mM ammonium acetate and 10 nM EDTA. The LC column was an Acquity UPLC Peptide BEH C18 column (50 × 2.1 mm; 1.7 μm; 130 Å). The gradient was 0 min, 0% B; 1 min, 0% B; 5 min, 40% B; 7.5 min, 64% B; 12.0 min, 64% B; 12.5 min, 82.5% B, 19 min, 85% B; 20 min, 95% B; 20.1 min, 0% B; 25 min, 0% B. The column flow rate was 0.3 mL/min, the autosampler temperature was 5 °C, the injection volume was 1 uL for mouse liver tissue and 2 μL for the other samples. The column temperature was 45 °C.

MS was performed on an AB Sciex TripleTOF 5600+ system (Q-TOF) equipped with a DuoSpray ion source. All analyses were performed in the high sensitivity mode for both TOF–MS and product ion scanning. Mass calibration was automatically performed every five injections using an APCI positive/negative calibration solution and a calibration delivery system (CDS). Data-dependent MS/MS acquisition (DDA) was used. The common parameters in both positive and negative ion mode were collision energy, 45 V; collision energy spread, 15 V; mass range, *m/z* 70–1250; curtain gas, 30; ion source gas 1, 50; ion source gas 2, 50; temperature, 500 °C for mouse ear tissue and 300 °C for the other samples; declustering potential, 80 V; RF transmission, default. The ion spray voltage floating in positive/negative ion mode was +5.5/–4.5 kV, respectively. The DDA parameters in both positive and negative ion mode were MS^1^ accumulation time, 250 ms; MS^2^ accumulation time, 100 ms; cycle time, 1300 ms; dependent product ion scan number, 10; intensity threshold, 100; exclusion time of precursor ion, 5 s; mass tolerance, 20 mDa; ignore peaks, within 6 Da; dynamic background subtraction, TRUE.

## Additional files

**Additional file 1**. **Figure** **S1** The fragment assignments of 12 sphingolipid classes. The annotations were combinatorially performed by hydrogen rearrangement rules in combination with substantial manual curation. The original spectra were obtained from LC/MS data of some biological samples including human cells, mouse tissues, and plant species.

**Additional file 2**. **Table S1**. Details of chemical descriptors for retention time predictions. **Table S2**. Details of lipid identification in HEK cells. **Table S3**. Details of lipid identification in HeLa cells. **Table S4**. Details of lipid identification in mouse liver tissue. **Table S5**. Details of lipid identification in mouse ear tissue. **Table S6**. Binary vector matrix as lipid descriptors used in hierarchical clustering analysis.

**Additional file 3**. **Figure** **S2**. The MS/MS annotations of ceramide [BS] in positive ion mode. The abbreviations of the hydrogen rearrangement rules follow our previous study [Ref. 9, Table 1].

**Additional file 4**. **Figure** **S3** Examples of lipid identifications for 12 sphingolipid classes and 9 glycerolipid classes in negative ion mode. The experimental- and in silico MS/MS spectra are shown in the upper (blue) and lower (red) panels, respectively. The ion abundances of in silico spectra were determined heuristically. The original spectra were obtained from LC/MS data of some biological samples including human cells, mouse tissues, and plant species.

**Additional file 5**. **Figure** **S4**. Details of hierarchical clustering analysis in Fig. 5c.
