# Supplementary material for: Comprehensive identification of sphingolipid species by in silico retention time and tandem mass spectral library
Source: J Cheminform. 2017 Mar 15;9:19. doi: 10.1186/s13321-017-0205-3 (PMC5352698; doi:10.1186/s13321-017-0205-3)
Supplement: Supplementary file 1 — Additional file 1. Figure S1 The fragment assignments of 12 sphingolipid classes. The annotations were combinatorially performed by hydrogen rearrangement rules in combination with substantial manual curation. The original spectra were obtained from LC/MS data of some biological samples including human cells, mouse tissues, and plant species. [file 13321_2017_205_MOESM1_ESM.pdf]

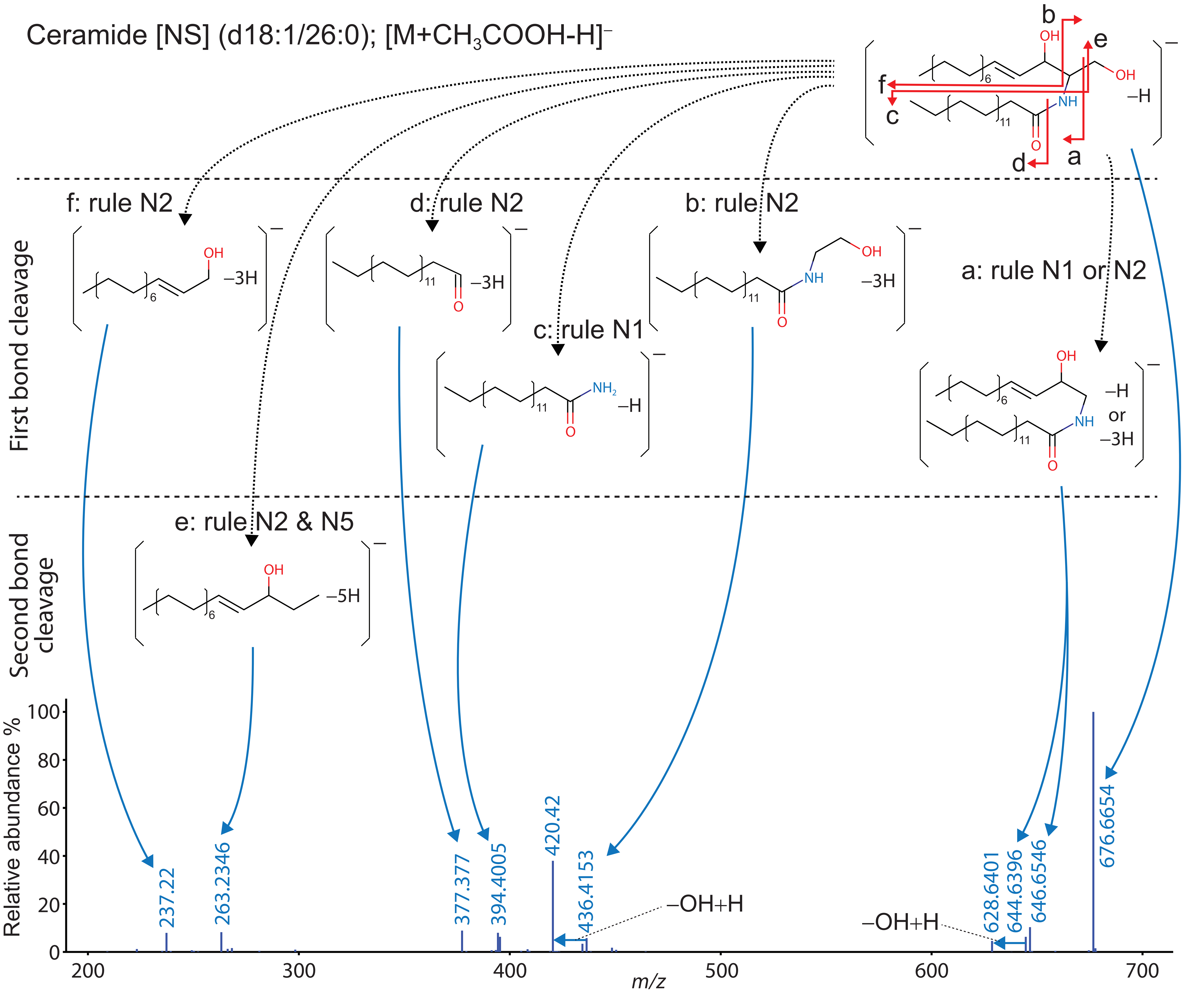

Ceramide [NS] (d18:1/26:0); [M+CH3COOH-H]⁻
b
e
f
c
d
a
First bond cleavage
f: rule N2
d: rule N2
b: rule N2
a: rule N1 or N2
c: rule N1
Second bond cleavage
e: rule N2 & N5
−3H
−H
−5H
or
Relative abundance %
m/z
237.22
263.2346
377.377
394.4005
420.42
436.4153
628.6401
644.6396
646.6546
676.6654
−OH+H
−OH+H

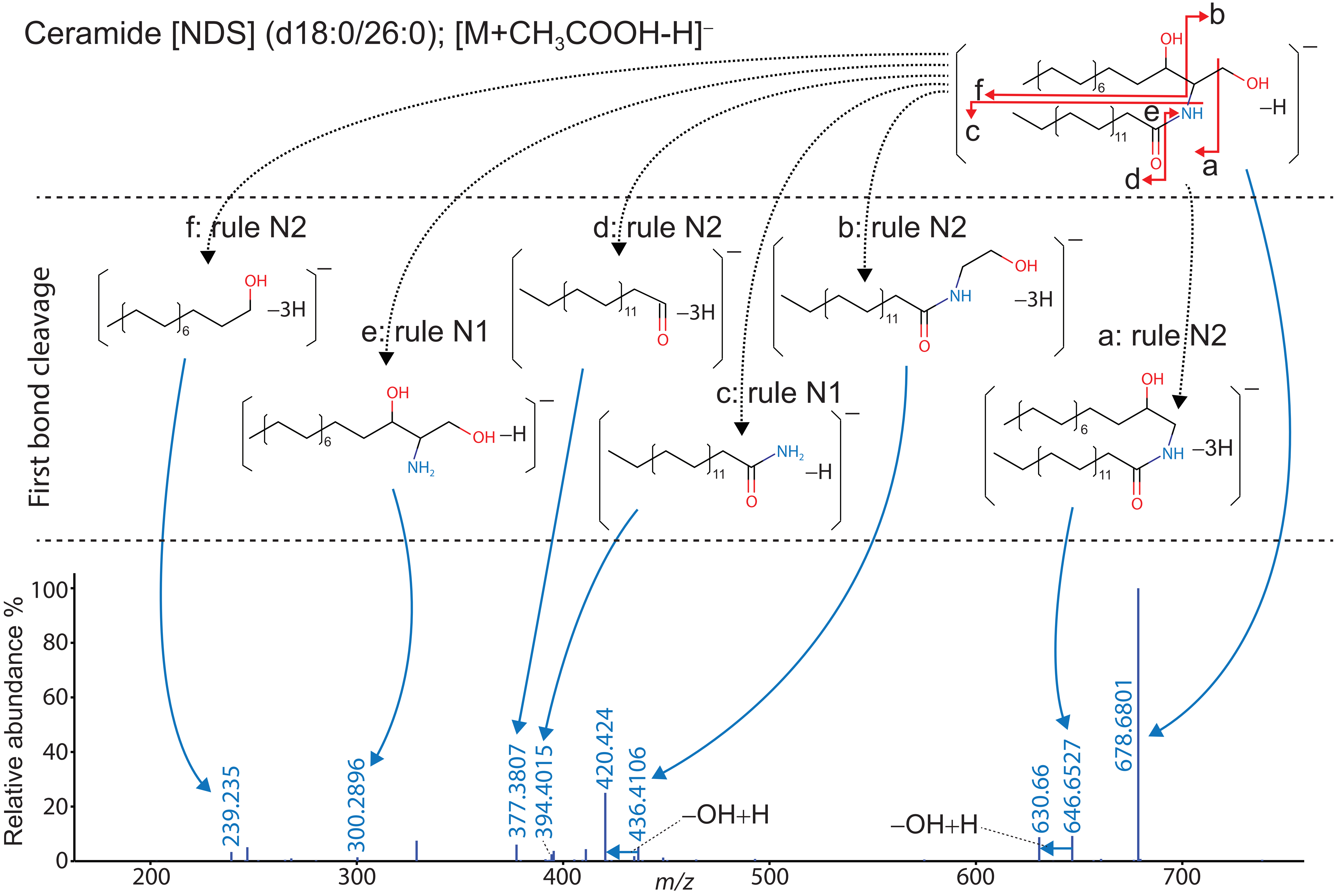

Ceramide [NDS] (d18:0/26:0); [M+CH3COOH-H]–
First bond cleavage
f: rule N2
e: rule N1
d: rule N2
c: rule N1
b: rule N2
a: rule N2
Relative abundance %
m/z
239.235
300.2896
377.3807
394.4015
420.424
436.4106
630.66
646.6527
678.6801
–OH+H
–OH+H

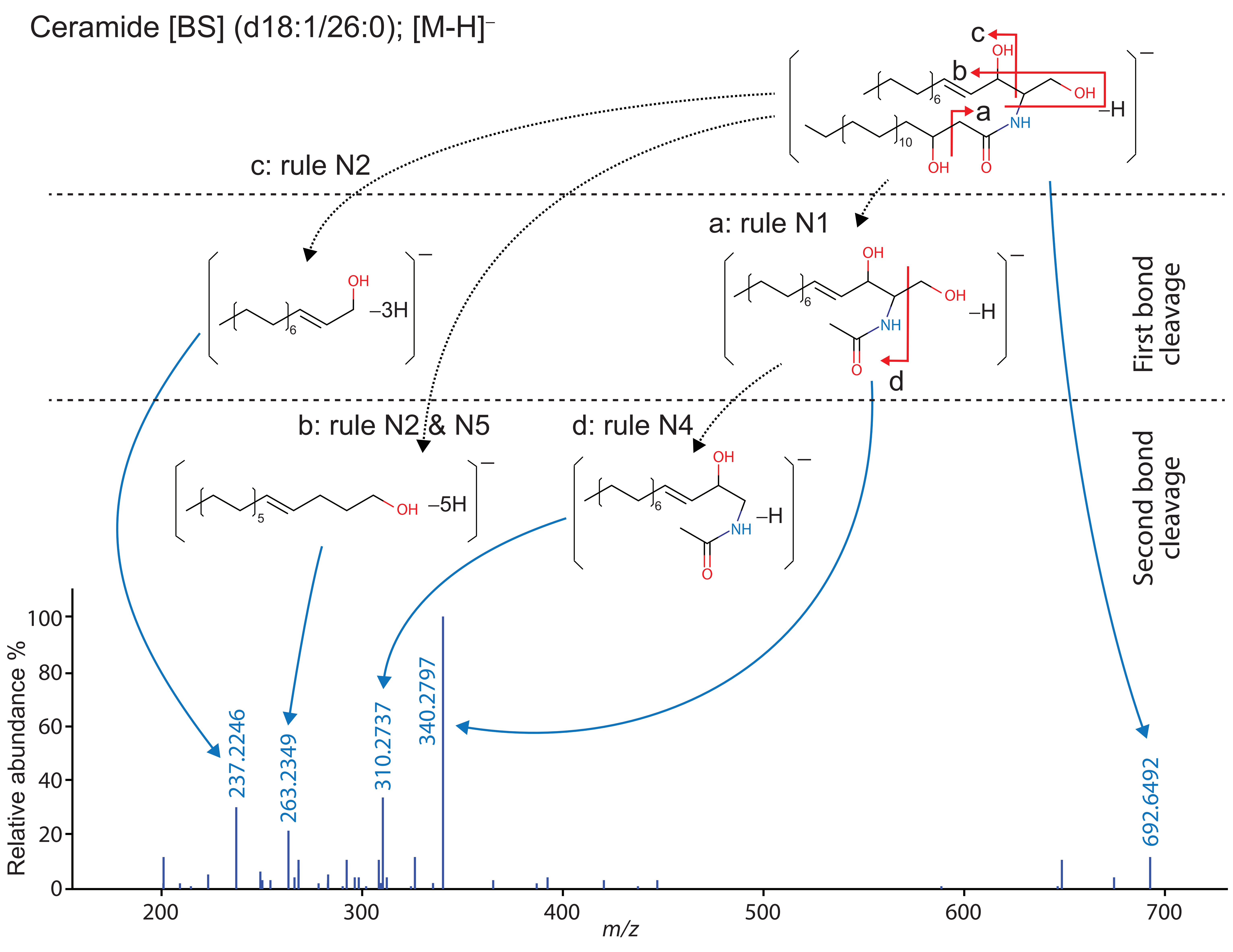

Ceramide [BS] (d18:1/26:0); [M-H]⁻
c: rule N2
a: rule N1
b: rule N2 & N5
d: rule N4
First bond cleavage
Second bond cleavage
–3H
–5H
–H
Relative abundance %
m/z
237.2246
263.2349
310.2737
340.2797
692.6492

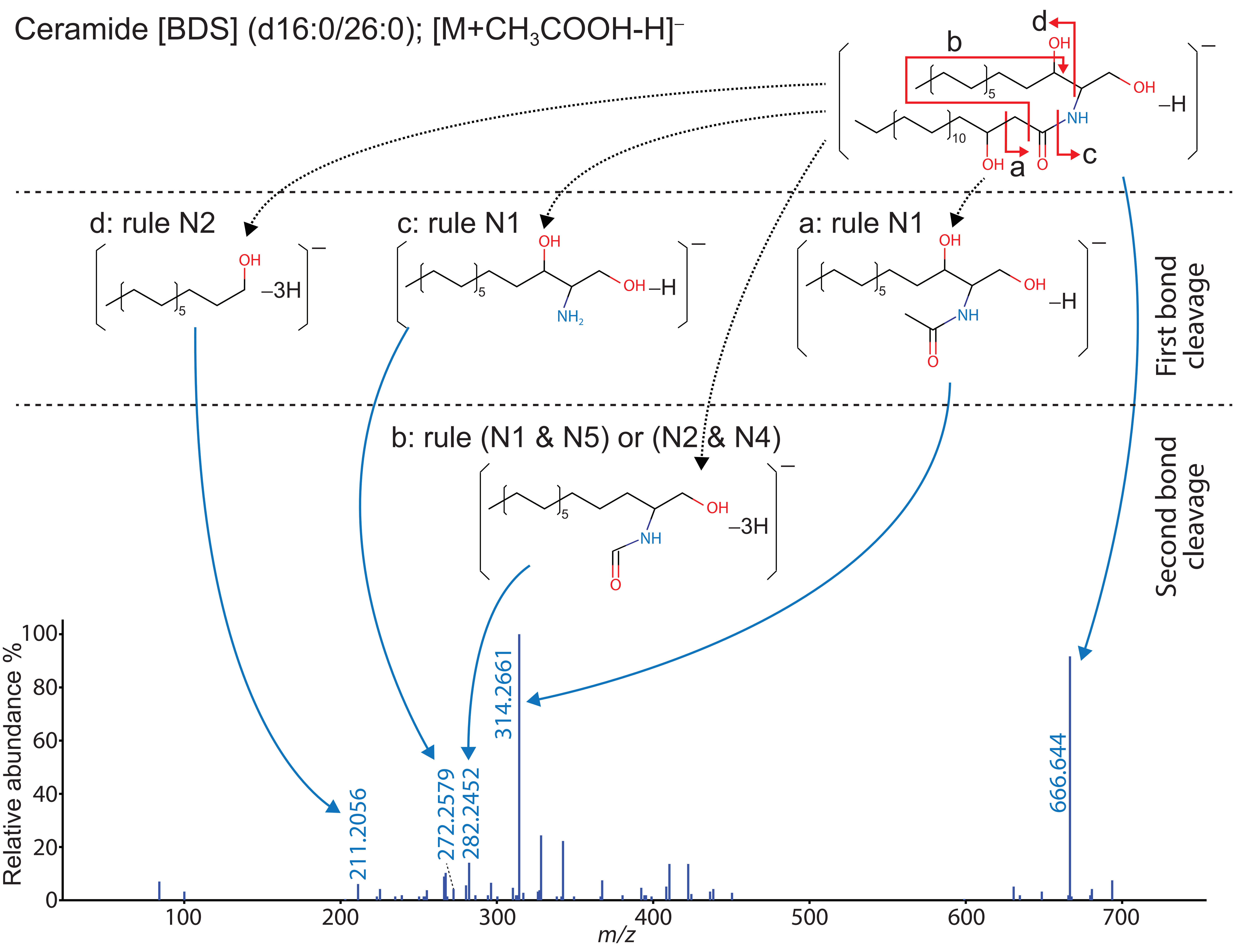
Ceramide [BDS] (d16:0/26:0); [M+CH3COOH-H]–
d
b
a
c
d: rule N2
c: rule N1
a: rule N1
b: rule (N1 & N5) or (N2 & N4)
First bond cleavage
Second bond cleavage
Relative abundance %
m/z
211.2056
272.2579
282.2452
314.2661
666.644

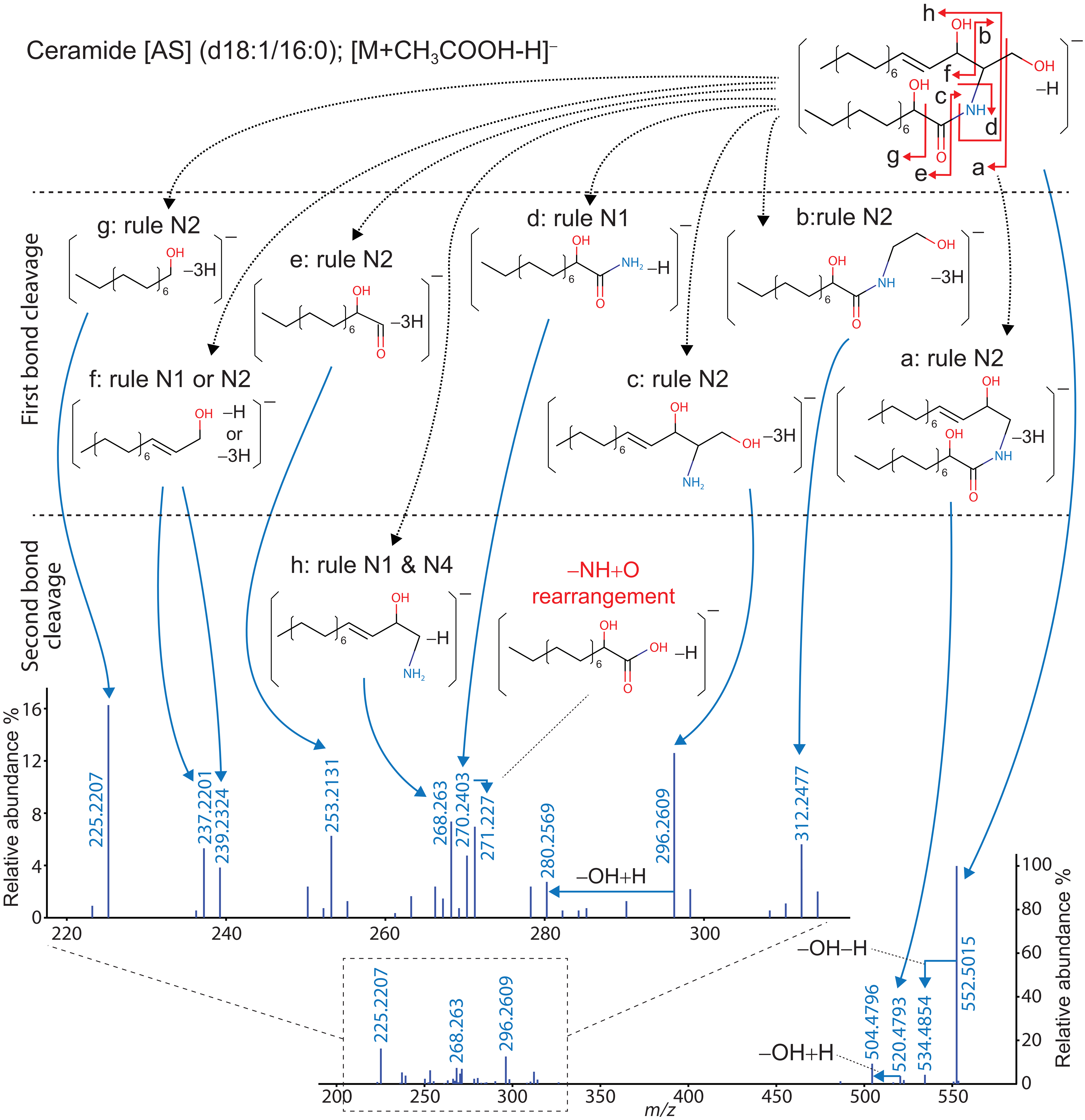

Ceramide [AS] (d18:1/16:0); [M+CH3COOH-H]−
First bond cleavage
Second bond cleavage
g: rule N2
e: rule N2
d: rule N1
b:rule N2
a: rule N2
c: rule N2
f: rule N1 or N2
h: rule N1 & N4
–NH+O rearrangement
–OH+H
–OH–H
Relative abundance %
m/z
225.2207
237.2201
239.2324
253.2131
268.263
270.2403
271.227
280.2569
296.2609
312.2477
504.4796
520.4793
534.4854
552.5015

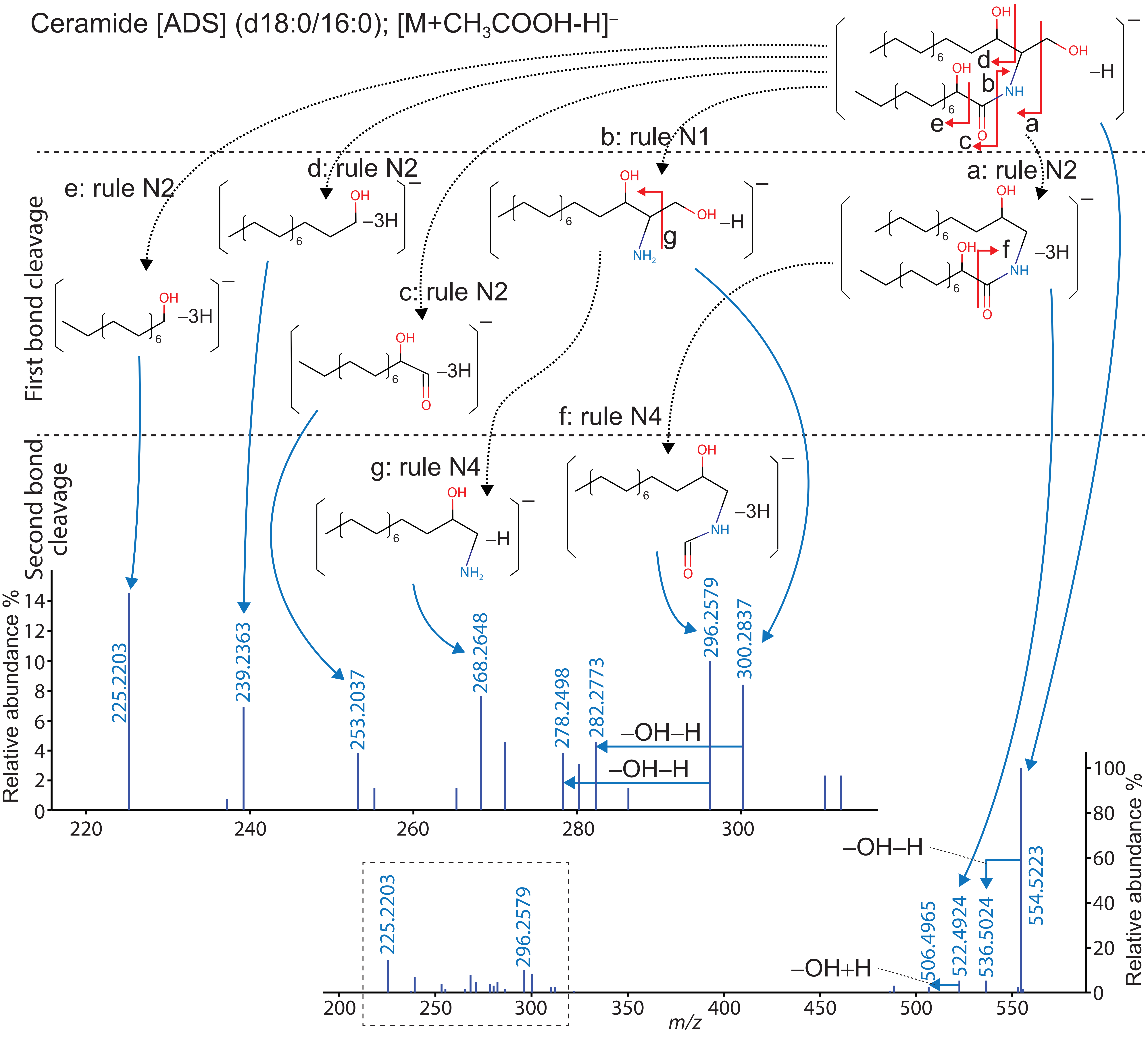

Ceramide [ADS] (d18:0/16:0); [M+CH3COOH-H]–
First bond cleavage
Second bond cleavage
a: rule N2
b: rule N1
c: rule N2
d: rule N2
e: rule N2
f: rule N4
g: rule N4
–OH–H
–OH–H
–OH–H
–OH+H
225.2203
239.2363
253.2037
268.2648
278.2498
282.2773
296.2579
300.2837
506.4965
522.4924
536.5024
554.5223
Relative abundance %
m/z

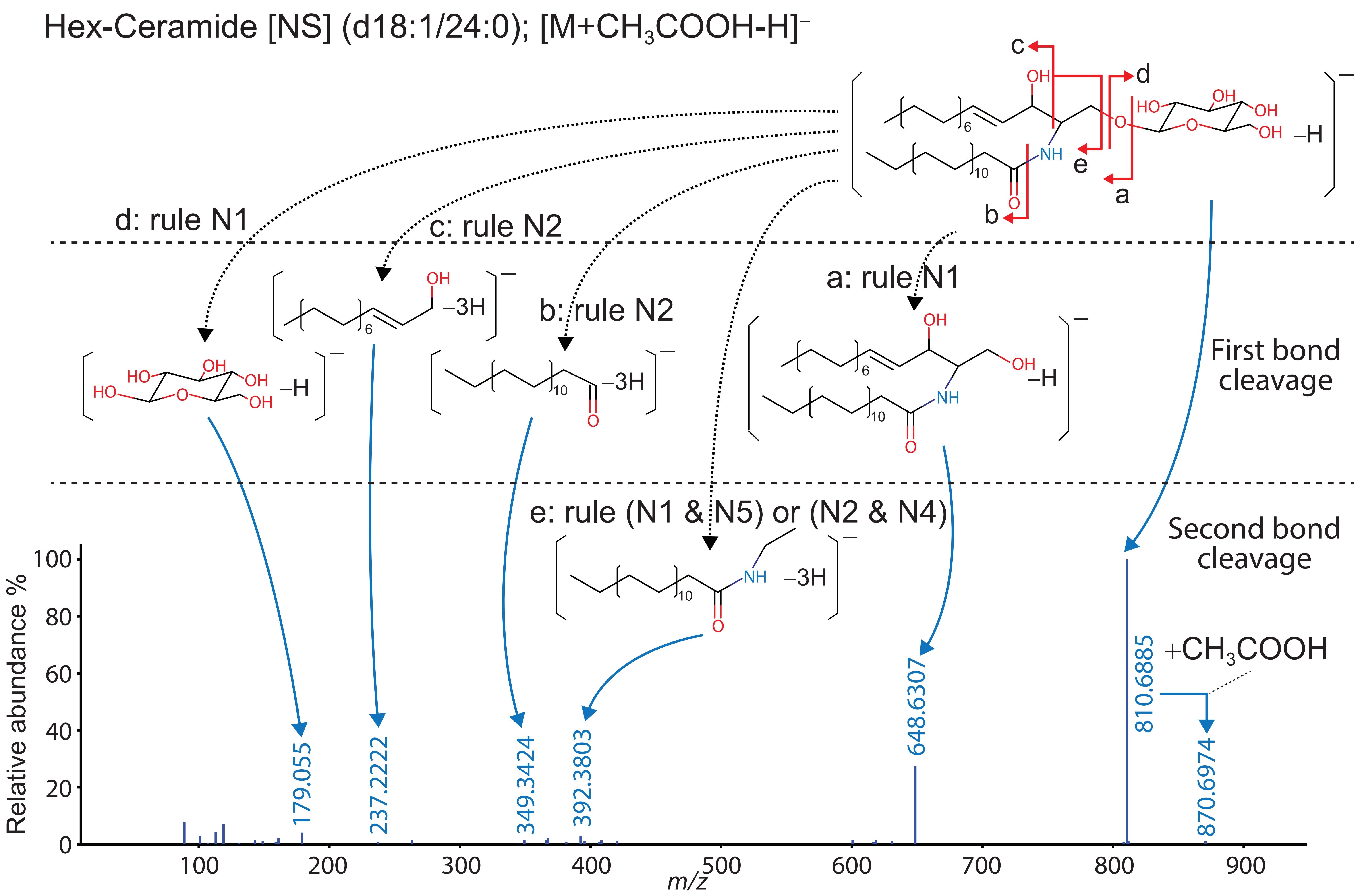
Hex-Ceramide [NS] (d18:1/24:0); [M+CH3COOH-H]–
d: rule N1
c: rule N2
b: rule N2
a: rule N1
e: rule (N1 & N5) or (N2 & N4)
First bond cleavage
Second bond cleavage
+CH3COOH
Relative abundance %
m/z
179.055
237.2222
349.3424
392.3803
648.6307
810.6885
870.6974

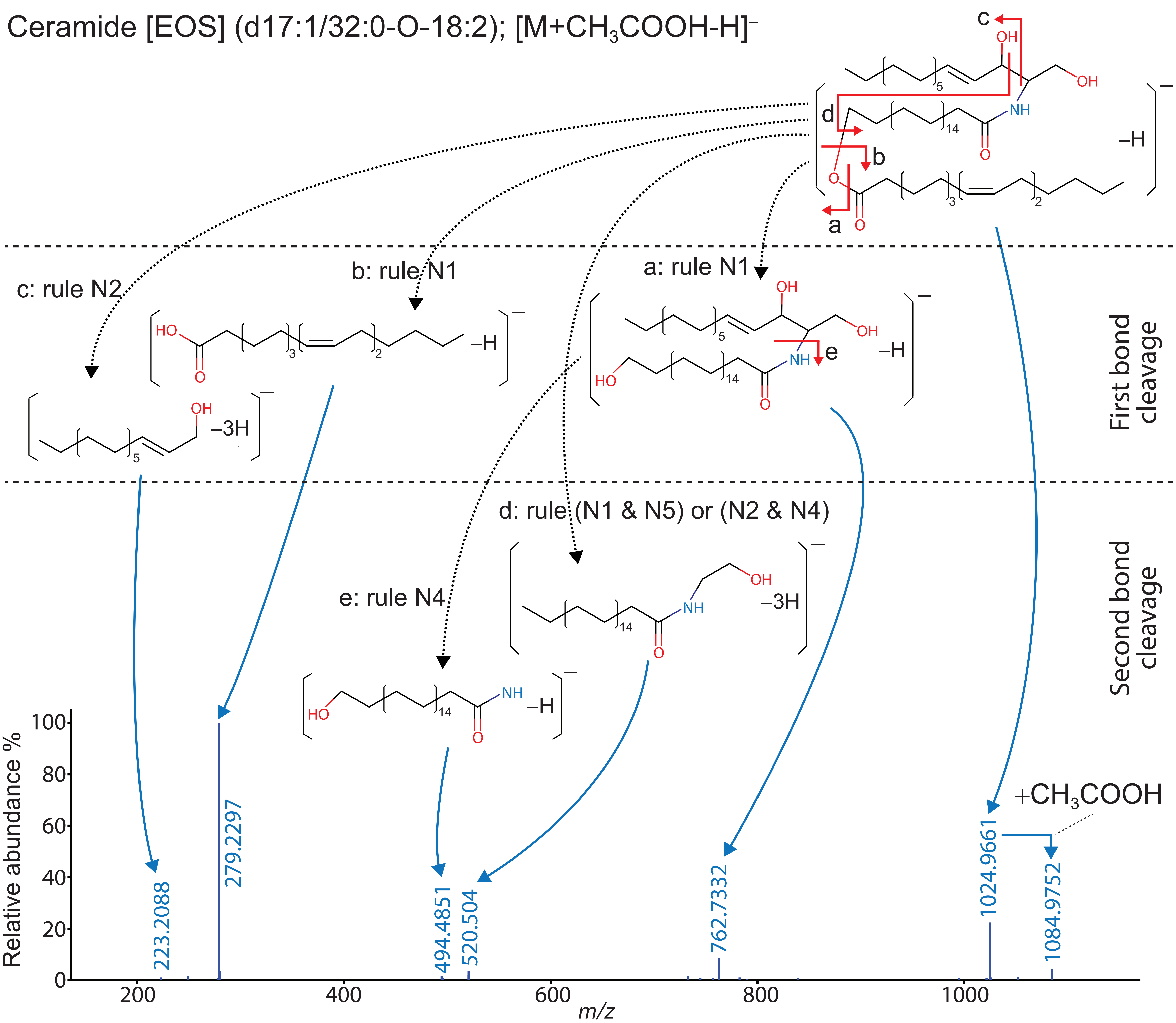

Ceramide [EOS] (d17:1/32:0-O-18:2); [M+CH3COOH-H]−
c: rule N2
b: rule N1
a: rule N1
d: rule (N1 & N5) or (N2 & N4)
e: rule N4
First bond cleavage
Second bond cleavage
+CH3COOH
Relative abundance %
m/z
223.2088
279.2297
494.4851
520.504
762.7332
1024.9661
1084.9752

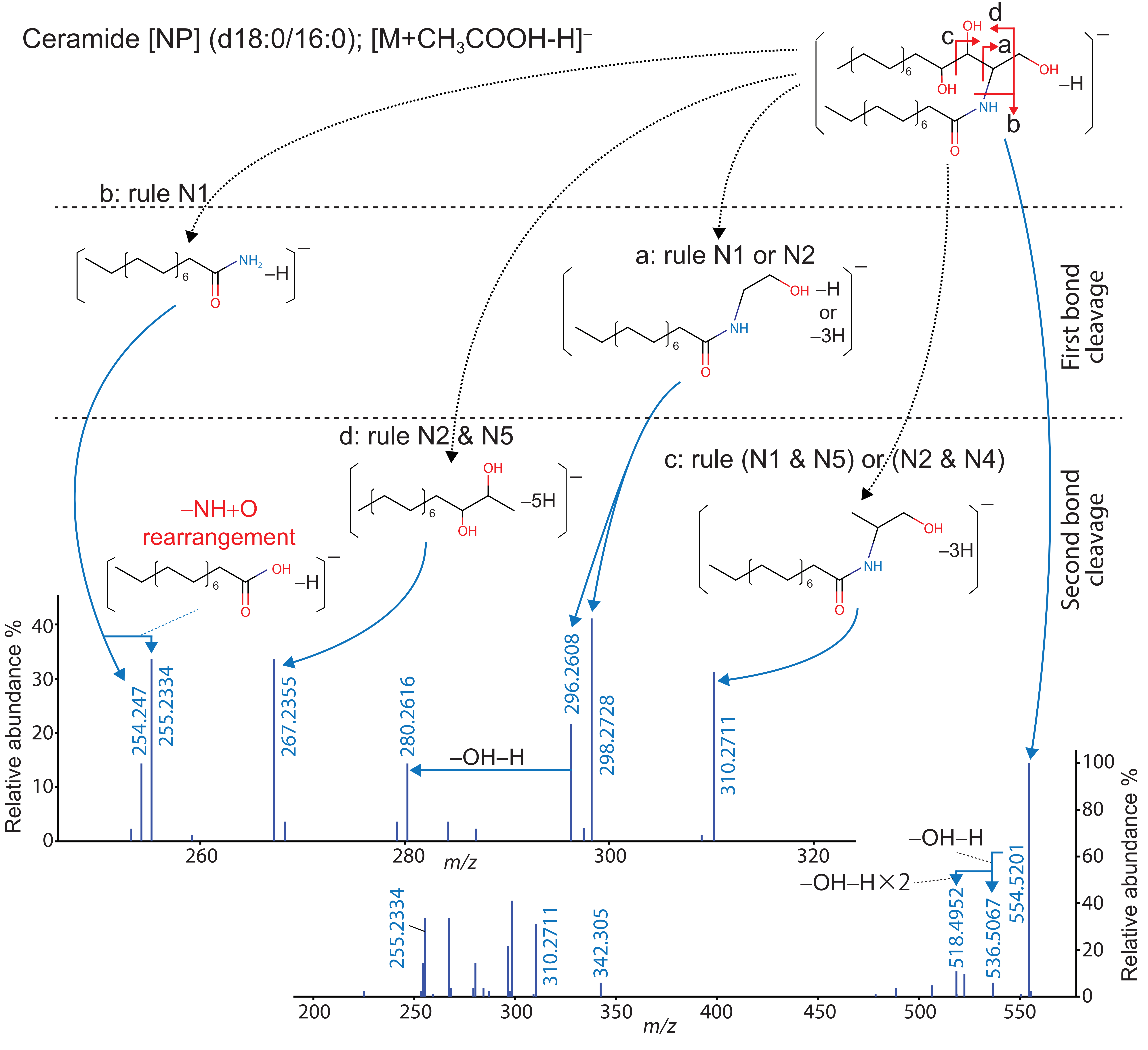

Ceramide [NP] (d18:0/16:0); [M+CH3COOH-H]⁻
b: rule N1
a: rule N1 or N2
d: rule N2 & N5
c: rule (N1 & N5) or (N2 & N4)
First bond cleavage
Second bond cleavage
–NH+O rearrangement
–OH–H
–OH–H×2
254.247
255.2334
267.2355
280.2616
296.2608
298.2728
310.2711
342.305
518.4952
536.5067
554.5201
Relative abundance %
m/z

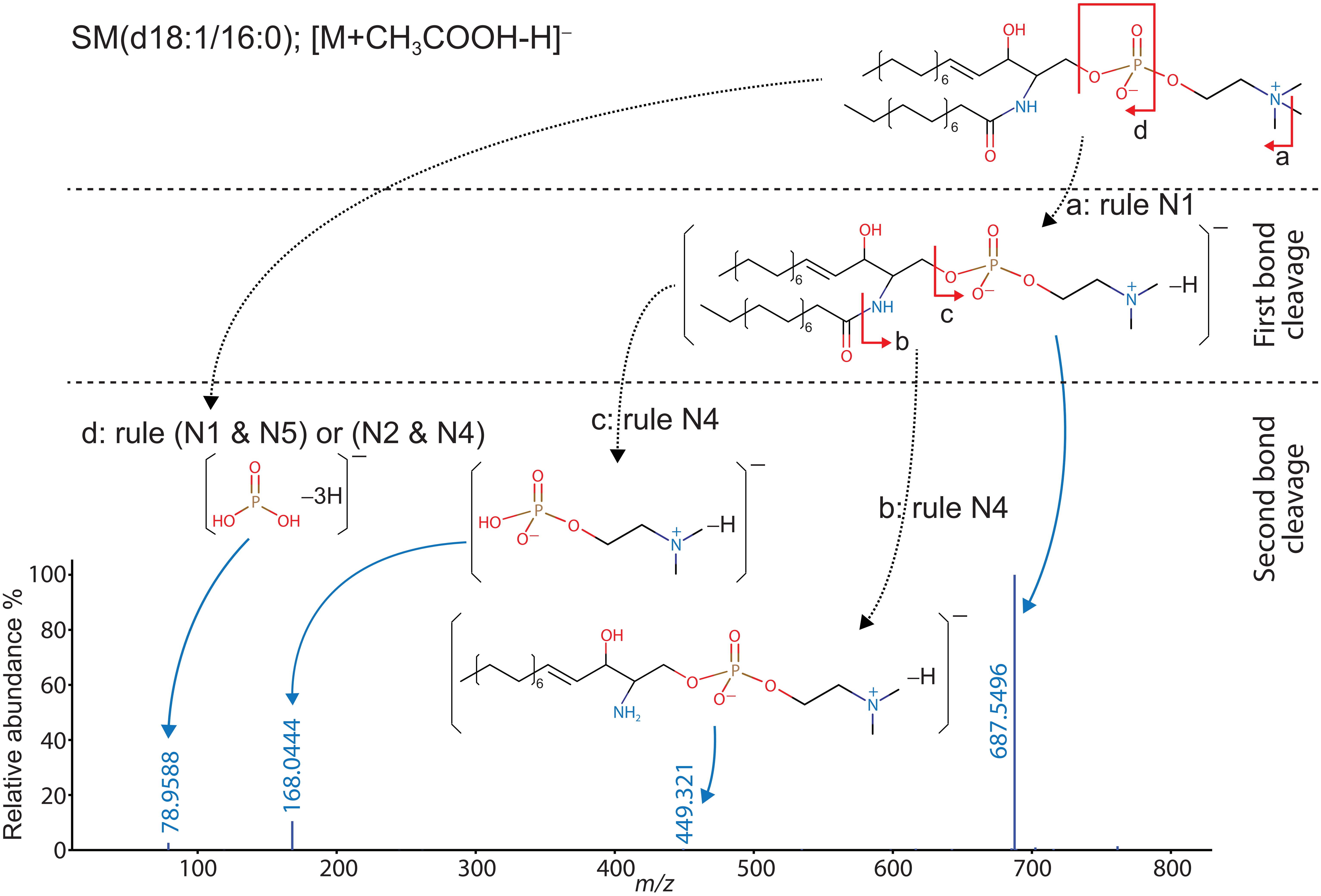

SM(d18:1/16:0); [M+CH3COOH-H]−
a: rule N1
First bond cleavage
Second bond cleavage
d: rule (N1 & N5) or (N2 & N4)
c: rule N4
b: rule N4
–3H
–H
Relative abundance %
m/z
78.9588
168.0444
449.321
687.5496

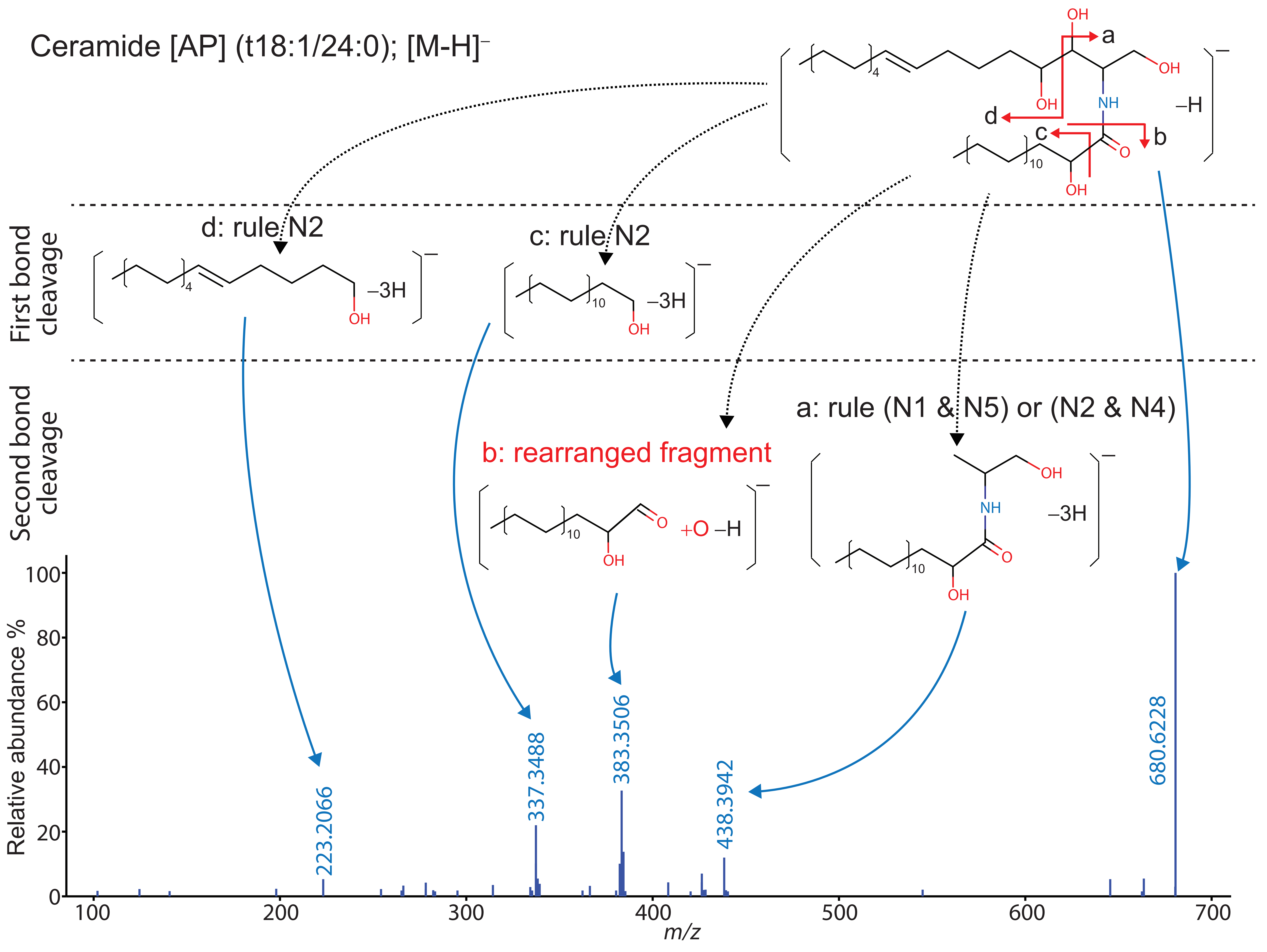

Ceramide [AP] (t18:1/24:0); [M-H]⁻
First bond cleavage
Second bond cleavage
d: rule N2
c: rule N2
b: rearranged fragment
a: rule (N1 & N5) or (N2 & N4)
Relative abundance %
m/z
223.2066
337.3488
383.3506
438.3942
680.6228

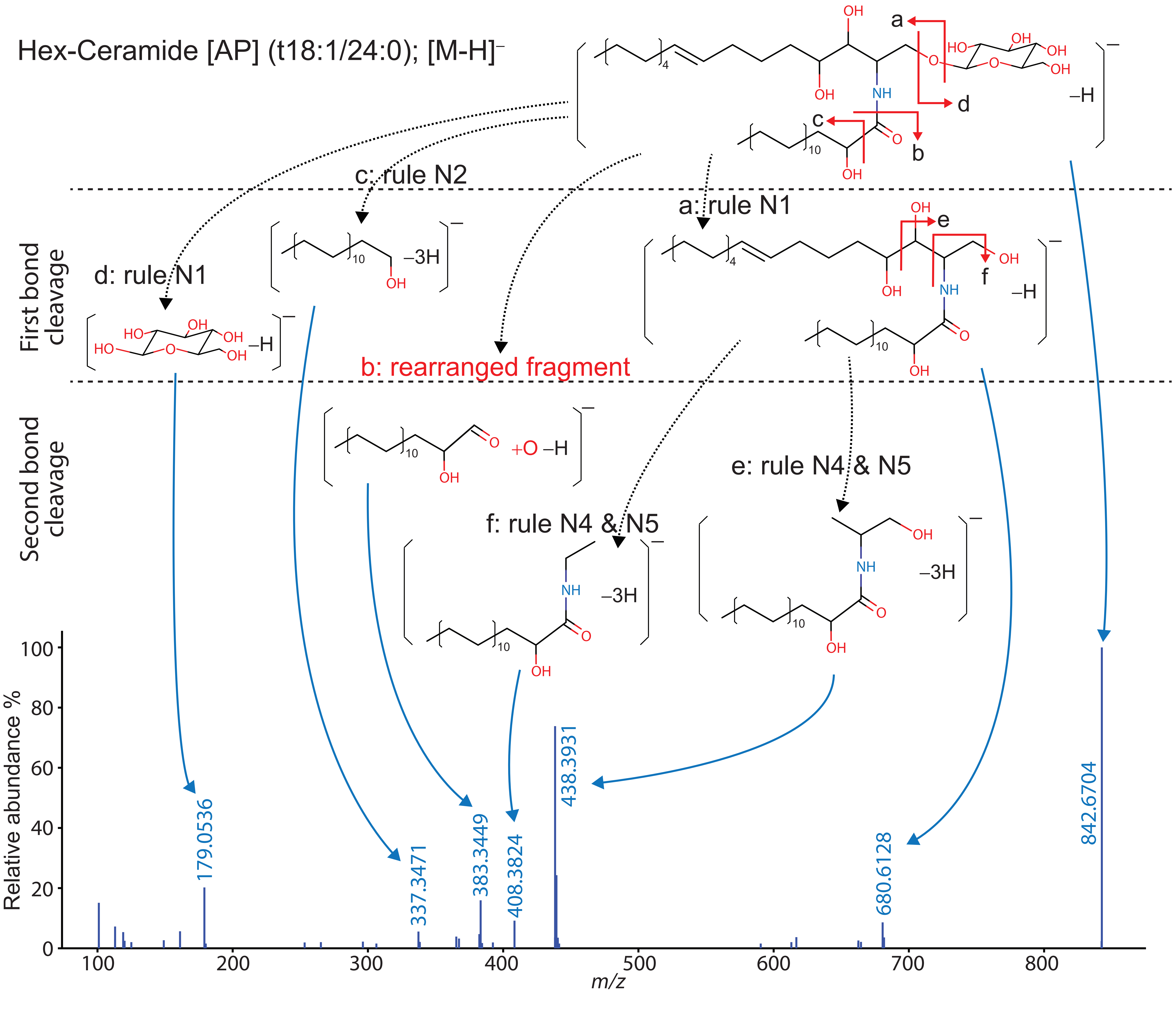

Hex-Ceramide [AP] (t18:1/24:0); [M-H]⁻
a
b
c
d
–H
First bond cleavage
c: rule N2
a: rule N1
d: rule N1
b: rearranged fragment
–3H
e
f
Second bond cleavage
+O –H
e: rule N4 & N5
f: rule N4 & N5
Relative abundance %
m/z
179.0536
337.3471
383.3449
408.3824
438.3931
680.6128
842.6704
