# Supplementary figures and images for: Comprehensive identification of sphingolipid species by in silico retention time and tandem mass spectral library
Source: J Cheminform. 2017 Mar 15;9:19. doi: 10.1186/s13321-017-0205-3 (PMC5352698; doi:10.1186/s13321-017-0205-3)

Ceramide [BS] (d17:1/24:0); [M+H]<sup>+</sup>  
 RT=13.127 min, *m/z*=652.6163

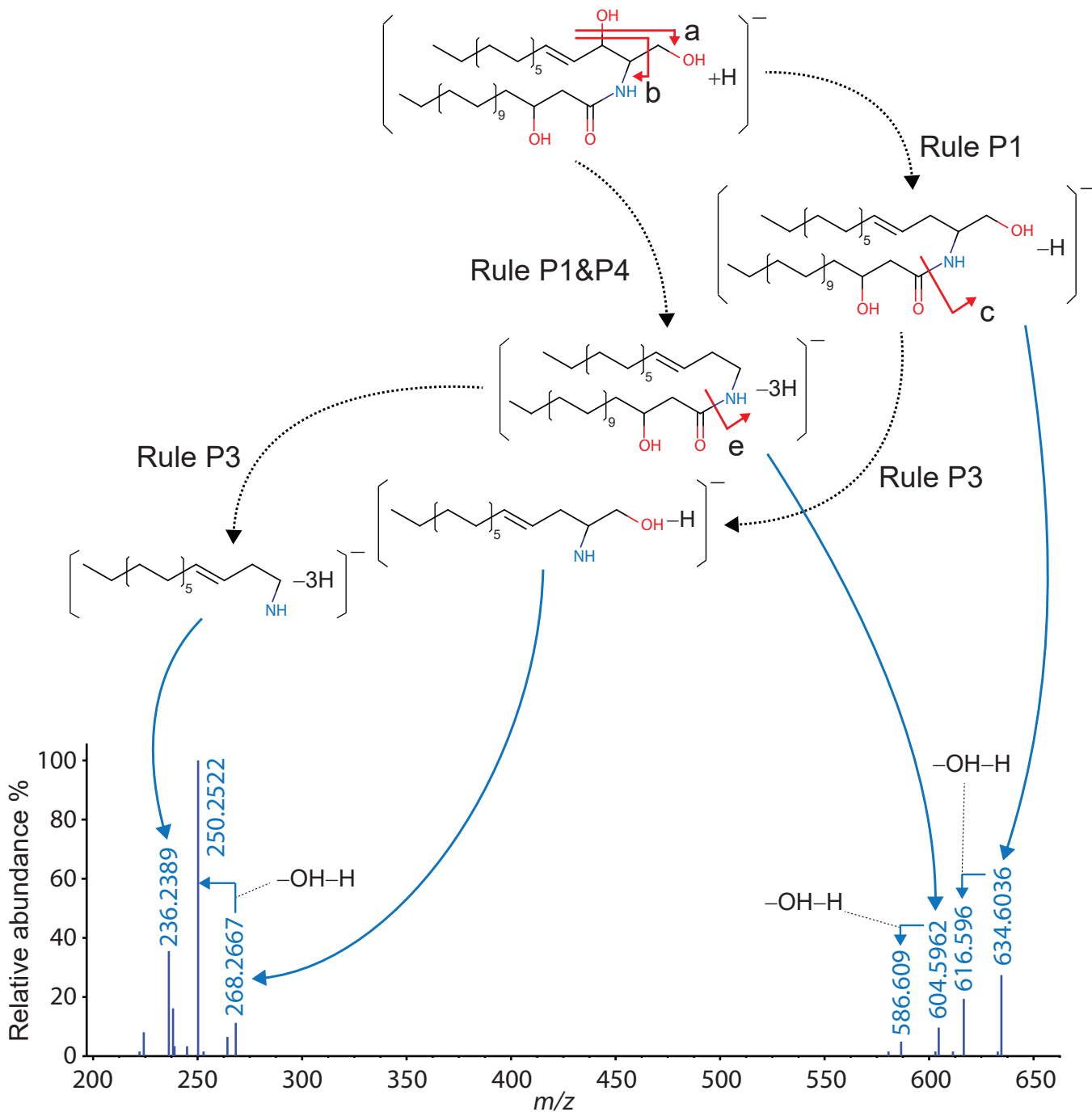

Supplement: Supplementary file 3 — Additional file 3. Figure S2. The MS/MS annotations of ceramide [BS] in positive ion mode. The abbreviations of the hydrogen rearrangement rules follow our previous study [Ref. 9, Table 1]. [file 13321_2017_205_MOESM3_ESM.pdf]
