# Supplementary material for: Comprehensive identification of sphingolipid species by in silico retention time and tandem mass spectral library
Source: J Cheminform. 2017 Mar 15;9:19. doi: 10.1186/s13321-017-0205-3 (PMC5352698; doi:10.1186/s13321-017-0205-3)

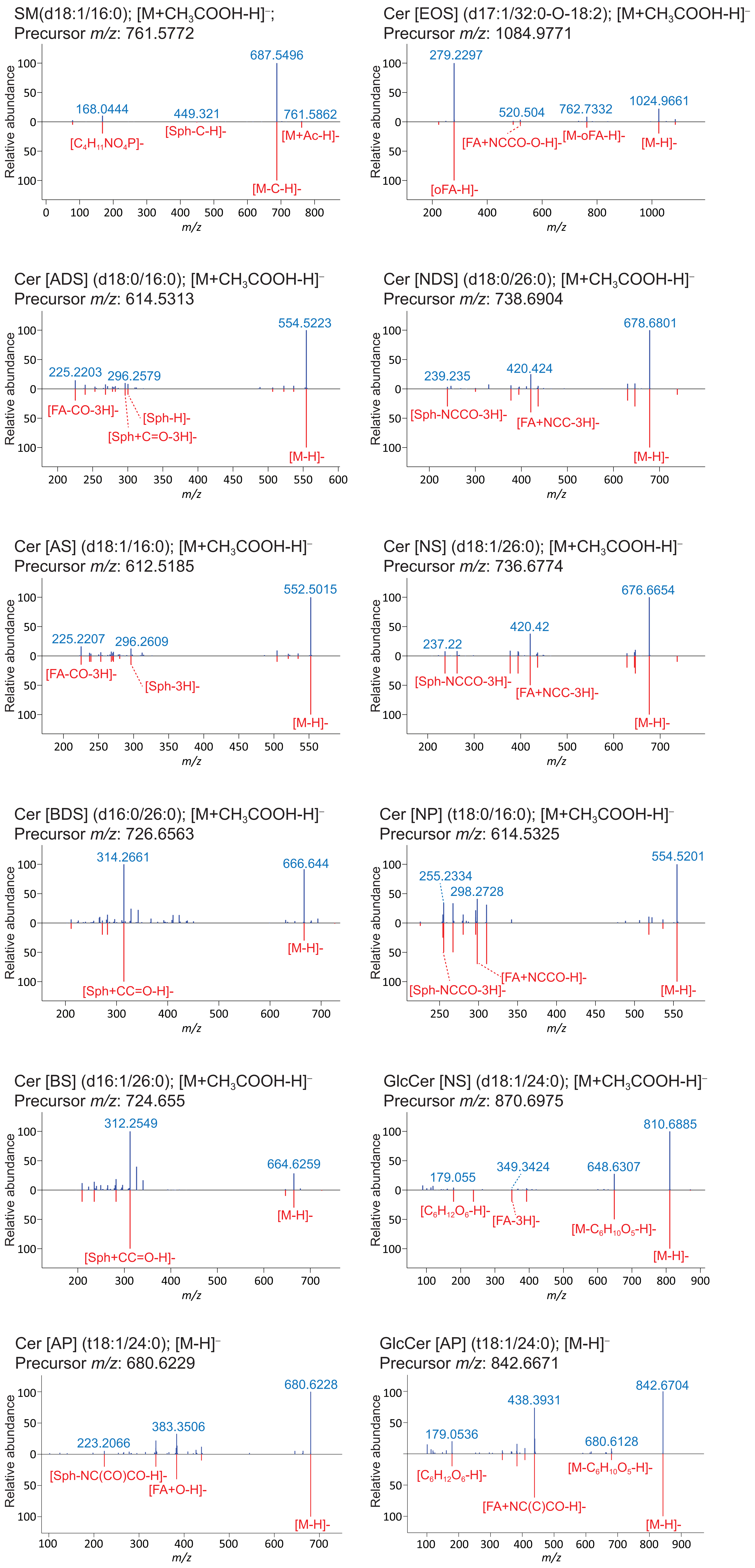
SM(d18:1/16:0); [M+CH3COOH-H]−;
Precursor m/z: 761.5772
168.0444
449.321
687.5496
761.5862
[C4H11NO4P]-
[Sph-C-H]-
[M+Ac-H]-
[M-C-H]-
Cer [EOS] (d17:1/32:0-O-18:2); [M+CH3COOH-H]−
Precursor m/z: 1084.9771
279.2297
520.504
762.7332
1024.9661
[FA+NCCO-O-H]-
[M-oFA-H]-
[M-H]-
[oFA-H]-
Cer [ADS] (d18:0/16:0); [M+CH3COOH-H]−
Precursor m/z: 614.5313
225.2203
296.2579
554.5223
[FA-CO-3H]-
[Sph-H]-
[Sph+C=O-3H]-
[M-H]-
Cer [NDS] (d18:0/26:0); [M+CH3COOH-H]−
Precursor m/z: 738.6904
239.235
420.424
678.6801
[Sph-NCCO-3H]-
[FA+NCC-3H]-
[M-H]-
Cer [AS] (d18:1/16:0); [M+CH3COOH-H]−
Precursor m/z: 612.5185
225.2207
296.2609
552.5015
[FA-CO-3H]-
[Sph-3H]-
[M-H]-
Cer [NS] (d18:1/26:0); [M+CH3COOH-H]−
Precursor m/z: 736.6774
237.22
420.42
676.6654
[Sph-NCCO-3H]-
[FA+NCC-3H]-
[M-H]-
Cer [BDS] (d16:0/26:0); [M+CH3COOH-H]−
Precursor m/z: 726.6563
314.2661
666.644
[M-H]-
[Sph+CC=O-H]-
Cer [NP] (t18:0/16:0); [M+CH3COOH-H]−
Precursor m/z: 614.5325
255.2334
298.2728
554.5201
[FA+NCCO-H]-
[Sph-NCCO-3H]-
[M-H]-
Cer [BS] (d16:1/26:0); [M+CH3COOH-H]−
Precursor m/z: 724.655
312.2549
664.6259
[M-H]-
[Sph+CC=O-H]-
GlcCer [NS] (d18:1/24:0); [M+CH3COOH-H]−
Precursor m/z: 870.6975
179.055
349.3424
648.6307
810.6885
[C6H12O6-H]-
[FA-3H]-
[M-C6H10O5-H]-
[M-H]-
Cer [AP] (t18:1/24:0); [M-H]−
Precursor m/z: 680.6229
223.2066
383.3506
680.6228
[Sph-NC(CO)CO-H]-
[FA+O-H]-
[M-H]-
GlcCer [AP] (t18:1/24:0); [M-H]−
Precursor m/z: 842.6671
179.0536
438.3931
680.6128
842.6704
[C6H12O6-H]-
[M-C6H10O5-H]-
[FA+NC(C)CO-H]-
[M-H]-
Relative abundance
m/z

LysoPC 18:2; $[M+CH_3COOH-H]^-$
Precursor *m/z*: 578.344

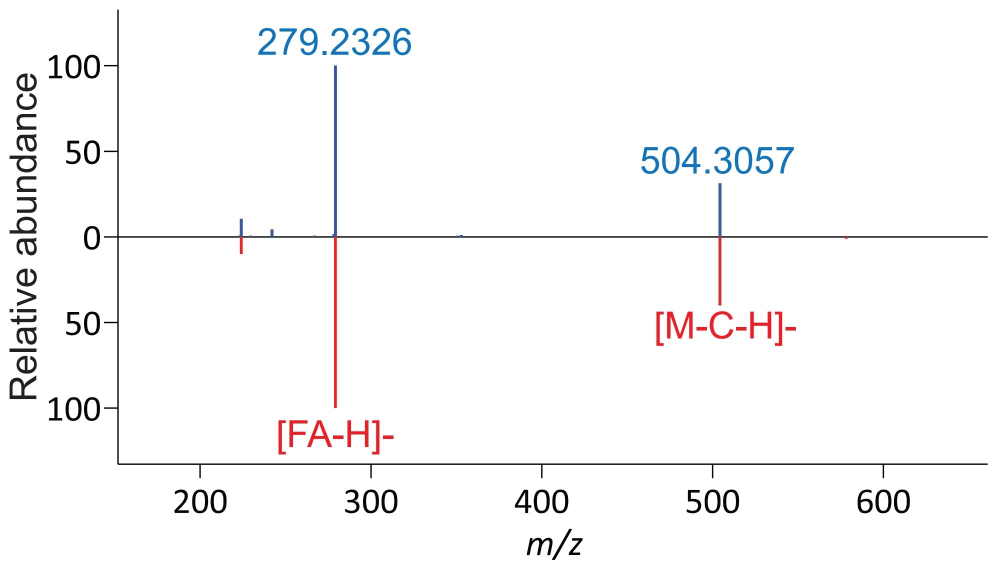

PI(18:0/16:1); $[M-H]^-$
Precursor *m/z*: 835.5283

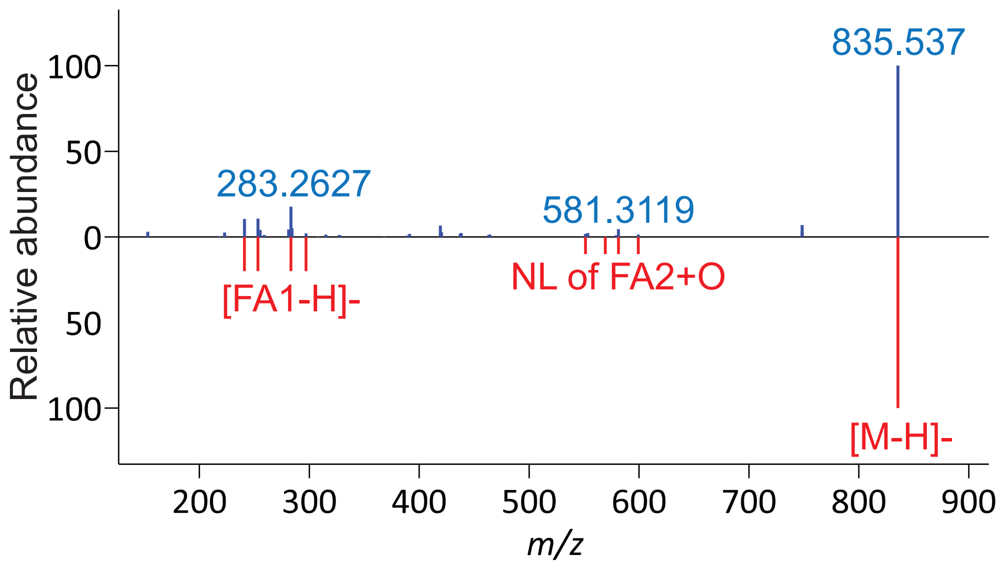

LysoPE 18:1; $[M-H]^-$
Precursor *m/z*: 478.2894

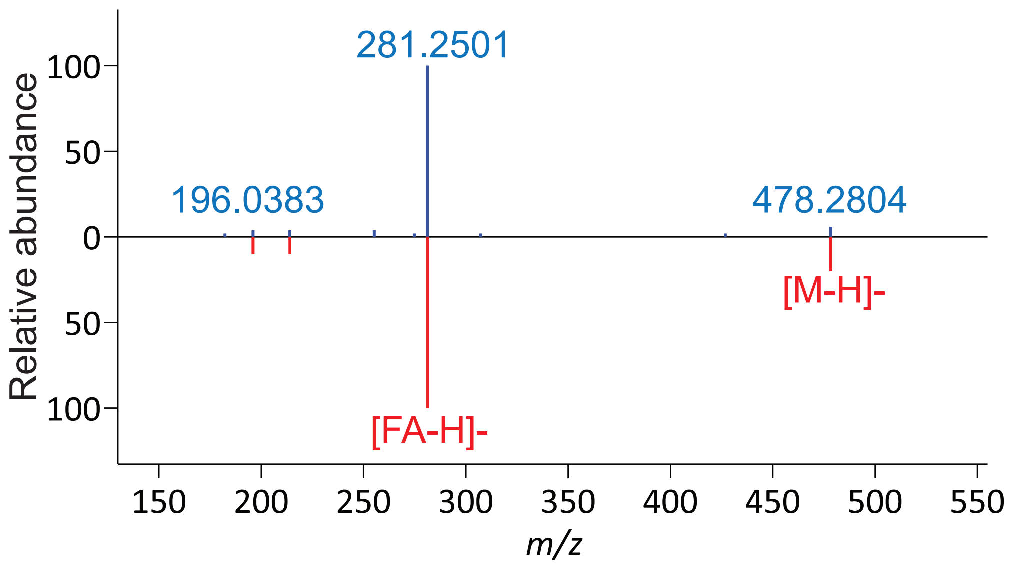

PS(18:0/22:5); $[M-H]^-$
Precursor *m/z*: 836.5422

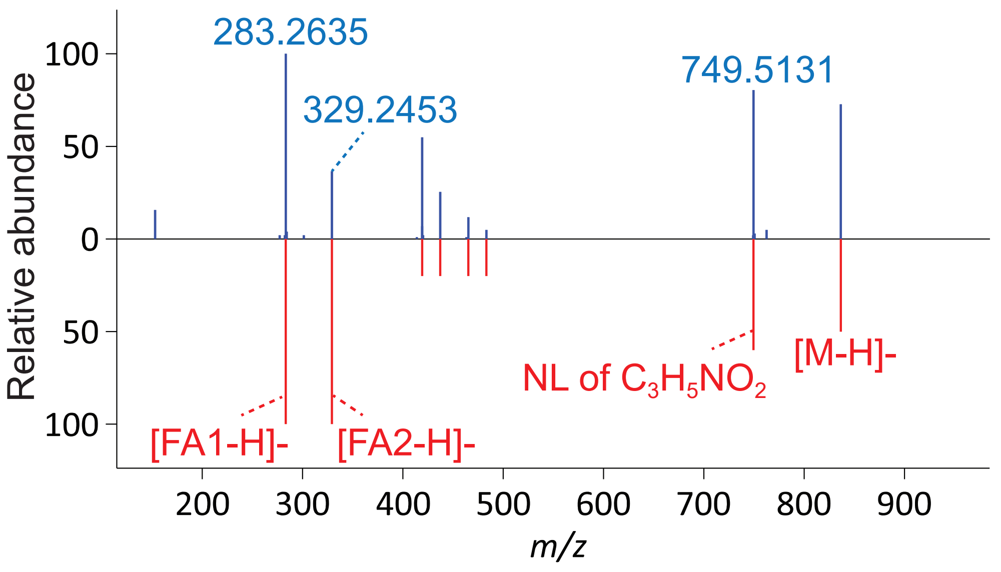

PC(16:0/20:4); $[M+CH_3COOH-H]^-$
Precursor *m/z*: 840.5733

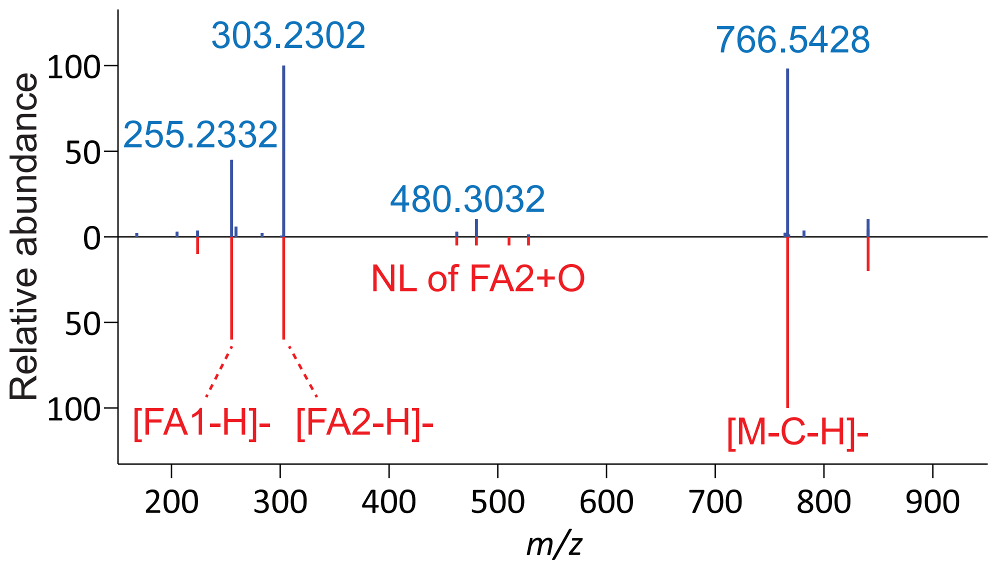

PC(e18:1/16:0); $[M+CH_3COOH-H]^-$
Precursor *m/z*: 804.6132

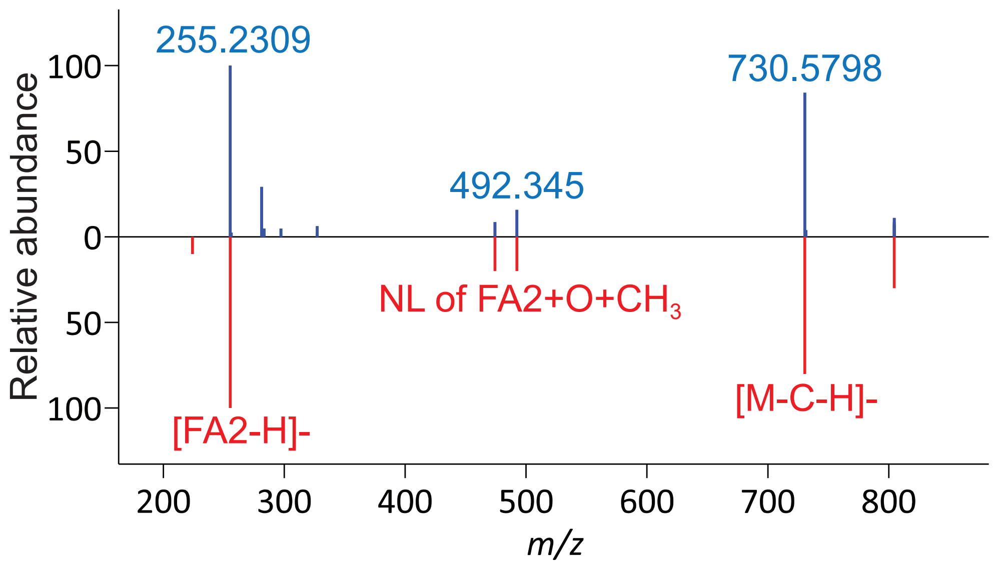

PE(18:0/22:4); $[M-H]^-$
Precursor *m/z*: 794.5637

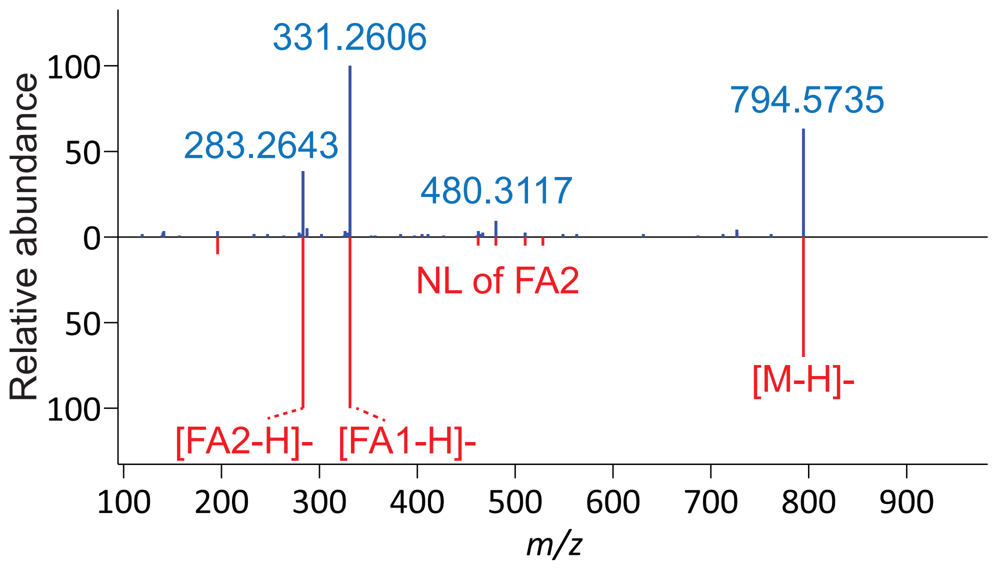

PE(e18:1/20:4); $[M-H]^-$
Precursor *m/z*: 750.5384

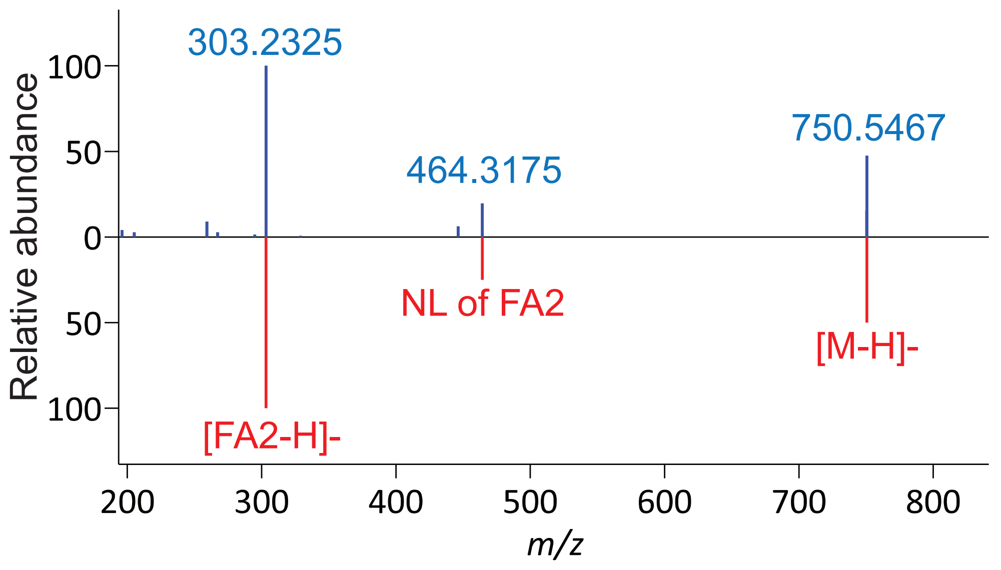

PG(16:0/18:1); $[M-H]^-$
Precursor *m/z*: 747.5227

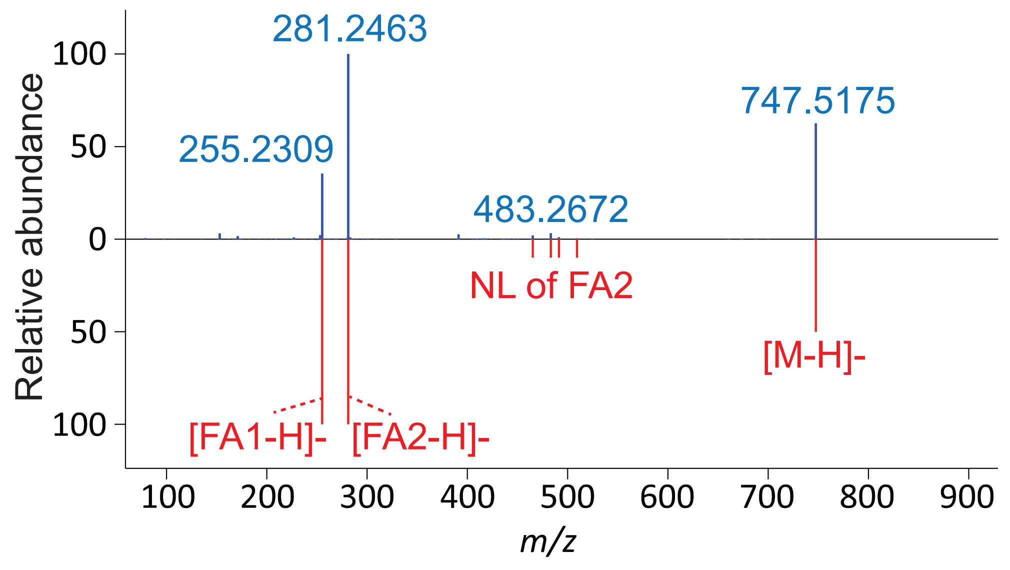

Supplement: Supplementary file 4 — Additional file 4. Figure S3 Examples of lipid identifications for 12 sphingolipid classes and 9 glycerolipid classes in negative ion mode. The experimental- and in silico MS/MS spectra are shown in the upper (blue) and lower (red) panels, respectively. The ion abundances of in silico spectra were determined heuristically. The original spectra were obtained from LC/MS data of some biological samples including human cells, mouse tissues, and plant species. [file 13321_2017_205_MOESM4_ESM.pdf]
