# Supplementary material for: Comprehensive identification of sphingolipid species by in silico retention time and tandem mass spectral library
Source: J Cheminform. 2017 Mar 15;9:19. doi: 10.1186/s13321-017-0205-3 (PMC5352698; doi:10.1186/s13321-017-0205-3)
Supplement: Supplementary file 5 — Additional file 5. Figure S4. Details of hierarchical clustering analysis in Fig. 5c. [file 13321_2017_205_MOESM5_ESM.pdf]

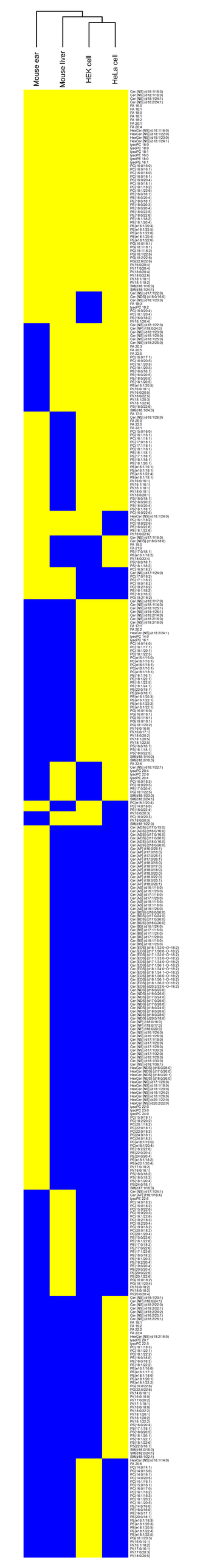
Mouse ear
Mouse liver
HEK cell
HeLa cell
Cer [NS] (d18:1/18:0)
Cer [NS] (d18:1/16:0)
Cer [NS] (d18:1/24:1)
Cer [NS] (d18:2/24:1)
FA 16:0
FA 16:1
FA 18:0
FA 18:1
FA 18:2
FA 20:3
FA 20:4
HexCer [NS] (d18:1/16:0)
HexCer [NS] (d18:1/22:0)
HexCer [NS] (d18:1/23:0)
HexCer [NS] (d18:1/24:1)
lysoPC 16:0
lysoPC 18:0
lysoPC 18:1
lysoPE 16:0
lysoPE 18:0
lysoPE 18:1
PC(16:0/16:0)
PC(16:0/16:1)
PC(16:0/18:0)
PC(16:0/18:1)
PC(16:0/20:4)
PC(18:1/18:2)
PC(18:1/18:2)
PC(18:1/22:6)
PE(16:0/18:1)
PE(16:0/20:4)
PE(16:0/18:1)
PE(16:0/20:3)
PE(18:0/22:5)
PE(18:0/22:5)
PE(18:0/22:6)
PE(18:1/18:2)
PE(18:1/20:4)
PE(16:0/22:5)
PE(16:1/22:5)
PE(16:1/22:6)
PE(18:1/20:4)
PE(18:1/22:6)
PG(16:0/18:1)
PG(18:1/16:0)
PG(18:1/18:2)
PG(18:1/22:6)
PG(22:6/22:6)
PI(16:0/20:4)
PI(18:0/18:1)
PI(18:0/20:4)
PI(18:0/22:6)
PI(18:1/18:1)
PI(18:1/18:2)
PS(18:0/22:6)
SM(d18:1/24:1)
FA 18:3
HexCer [NS] (d18:1/26:0)
FA 20:0
FA 22:0
FA 22:1
PC(15:0/16:0)
PC(16:1/16:1)
PC(16:1/18:1)
PC(17:0/18:1)
PC(17:1/18:1)
PC(18:1/18:1)
PC(16:1/18:1)
PC(18:1/18:1)
PE(18:1/20:1)
PE(16:1/16:1)
PE(a16:1/18:1)
PE(a16:1/18:1)
PE(a16:1/22:4)
PE(a18:1/18:1)
PE(a18:1/20:4)
PI(16:1/16:1)
PI(16:1/18:1)
PI(18:0/18:1)
PI(18:0/20:1)
PI(18:0/20:3)
PS(18:0/20:3)
PS(18:0/20:4)
PS(18:1/18:1)
PC(16:0/22:6)
HexCer [NS] (d18:1/24:0)
PC(16:1/18:2)
PC(16:1/22:6)
PE(16:0/22:6)
PE(18:1/22:6)
PI(16:0/22:6)
Cer [NS] (d17:1/16:0)
HexCer [NS] (d18:0/18:0)
FA 19:0
FA 21:0
PE(17:0/18:1)
PE(a16:1/18:2)
PE(16:1/22:6)
PS(16:0/18:1)
PS(18:1/18:2)
PC(16:0/18:2)
Cer [NS] (d17:1/24:0)
PC(17:1/18:2)
PC(18:1/18:2)
PC(18:2/18:2)
PE(16:1/18:2)
PE(18:2/18:2)
PG(18:2/18:2)
HexCer [NS] (d18:1/17:0)
Cer [NS] (d18:1/14:0)
Cer [NS] (d18:1/25:1)
Cer [NS] (d18:1/26:1)
Cer [NS] (d18:2/14:0)
Cer [NS] (d18:2/16:0)
Cer [NS] (d18:2/18:0)
FA 20:1
FA 20:2
HexCer [NS] (d18:2/24:1)
lysoPC 14:0
lysoPC 16:1
lysoPC 18:2
PC(16:1/17:1)
PC(18:1/20:1)
PC(18:1/22:5)
PC(a16:1/16:0)
PC(a16:1/16:1)
PC(a16:1/18:1)
PC(a18:1/18:1)
PE(16:1/16:1)
PE(18:1/22:1)
PE(18:1/22:5)
PE(18:1/22:6)
PE(22:5/18:1)
PE(24:0/18:1)
PE(a16:1/20:3)
PE(a16:1/22:1)
PE(a18:1/22:1)
PG(16:0/16:0)
PG(16:0/16:1)
PG(16:1/18:1)
PG(18:0/18:1)
PG(18:1/20:2)
PI(16:0/18:1)
PI(16:0/17:1)
PI(18:0/20:2)
PI(18:1/20:5)
PI(18:1/22:5)
PS(16:0/18:1)
PS(16:1/18:1)
PS(18:0/22:5)
SM(d18:1/14:0)
SM(d18:2/16:0)
FA 22:6
Cer [NS] (d18:1/22:1)
FA 20:5
lysoPC 22:6
lysoPE 20:4
PC(16:0/18:3)
PC(18:0/20:5)
PC(18:0/22:6)
PG(18:1/22:5)
SM(d18:1/23:0)
SM(d18:2/24:1)
PC(a16:1/20:4)
PC(14:0/18:0)
PC(18:0/22:4)
PC(18:0/20:4)
PC(18:0/20:3)
SM(d18:1/22:0)
Cer [ADS] (d17:0/15:0)
Cer [ADS] (d17:1/15:0)
Cer [ADS] (d17:1/16:0)
Cer [ADS] (d17:0/26:0)
Cer [ADS] (d18:0/16:0)
Cer [ADS] (d18:0/26:0)
Cer [AP] (d18:0/26:1)
Cer [AP] (d17:0/25:1)
Cer [AP] (d17:0/26:1)
Cer [AP] (d18:0/16:0)
Cer [AP] (d18:0/17:0)
Cer [AP] (d18:0/18:0)
Cer [AP] (d18:0/22:0)
Cer [AP] (d18:0/23:1)
Cer [AP] (d18:0/26:1)
Cer [AS] (d16:1/16:0)
Cer [AS] (d17:1/16:0)
Cer [AS] (d17:1/18:0)
Cer [AS] (d17:1/26:0)
Cer [AS] (d18:1/18:0)
Cer [AS] (d18:1/18:0)
Cer [AS] (d18:1/26:0)
Cer [BDS] (d16:0/26:0)
Cer [BDS] (d17:0/26:0)
Cer [BDS] (d17:0/26:0)
Cer [BDS] (d18:0/26:0)
Cer [BS] (d16:1/24:0)
Cer [BS] (d17:1/16:0)
Cer [BS] (d17:1/24:0)
Cer [BS] (d17:1/26:0)
Cer [BS] (d18:1/16:0)
Cer [BS] (d18:1/26:0)
Cer [EOS] (d16:1/32:0-O-18:2)
Cer [EOS] (d17:1/30:0-O-18:2)
Cer [EOS] (d17:1/32:0-O-18:2)
Cer [EOS] (d17:1/34:0-O-18:2)
Cer [EOS] (d17:1/34:1-O-18:2)
Cer [EOS] (d18:1/34:1-O-18:2)
Cer [EOS] (d18:1/34:2-O-18:2)
Cer [EOS] (d18:1/36:1-O-18:2)
Cer [EOS] (d18:1/36:2-O-18:2)
Cer [EOS] (d20:2/32:0-O-18:2)
Cer [NDS] (d16:0/25:0)
Cer [NDS] (d16:0/26:0)
Cer [NDS] (d17:0/24:0)
Cer [NDS] (d17:0/26:0)
Cer [NDS] (d17:0/28:0)
Cer [NDS] (d18:0/24:0)
Cer [NDS] (d18:0/26:0)
Cer [NDS] (d18:0/28:0)
Cer [NDS] (d18:0/30:0)
Cer [NP] (d18:0/16:0)
Cer [NP] (d18:0/17:0)
Cer [NP] (d18:0/20:0)
Cer [NS] (d16:1/24:0)
Cer [NS] (d17:1/16:0)
Cer [NS] (d17:1/18:0)
Cer [NS] (d17:1/26:0)
Cer [NS] (d17:1/28:0)
Cer [NS] (d17:1/30:0)
Cer [NS] (d17:1/32:0)
Cer [NS] (d18:1/28:0)
Cer [NS] (d18:1/30:0)
Cer [NS] (d18:1/36:1)
HexCer [NDS] (d16:0/28:0)
HexCer [NDS] (d17:0/26:0)
HexCer [NDS] (d18:0/20:1)
HexCer [NDS] (d18:0/24:0)
HexCer [NS] (d17:1/26:0)
HexCer [NS] (d18:1/18:0)
HexCer [NS] (d18:1/20:0)
HexCer [NS] (d18:1/24:2)
HexCer [NS] (d18:1/26:0)
HexCer [NS] (d20:1/22:0)
HexCer [NS] (d20:2/22:0)
lysoPC 23:0
lysoPC 23:0
lysoPC 24:0
PC(15:0/18:1)
PC(16:0/18:2)
PC(20:1/18:2)
PC(22:0/18:1)
PC(22:0/18:2)
PC(24:0/18:1)
PC(24:0/18:2)
PC(a16:1/16:0)
PC(a18:1/18:1)
PE(18:2/22:6)
PE(22:0/20:4)
PE(24:0/20:4)
PE(a18:1/18:2)
PE(a18:1/22:6)
PI(17:0/18:2)
PI(18:0/16:1)
PI(18:0/18:2)
PI(18:0/18:2)
PI(18:0/20:4)
PS(24:0/18:1)
SM(d17:1/16:0)
Cer [NS] (d17:1/24:1)
Cer [AP] (d18:1/18:4)
lysoPE 22:6
PC(14:0/18:2)
PC(15:0/18:2)
PC(15:0/22:6)
PC(16:0/20:3)
PC(16:1/22:6)
PC(18:2/18:3)
PC(18:2/20:4)
PC(19:0/18:2)
PC(20:0/18:2)
PC(20:1/20:4)
PE(15:0/22:6)
PE(16:1/22:6)
PE(17:0/18:2)
PE(17:1/18:2)
PE(17:1/22:6)
PE(18:0/18:2)
PE(18:1/20:3)
PE(18:2/20:4)
PE(20:0/20:4)
PE(20:0/20:4)
PE(20:0/22:6)
PE(20:1/22:6)
PE(16:0/18:2)
PE(18:1/20:4)
PI(16:0/18:2)
PI(18:1/18:2)
PI(20:0/20:4)
Cer [NS] (d18:1/23:1)
Cer [NP] (d18:0/24:1)
Cer [NS] (d18:2/22:0)
Cer [NS] (d18:2/23:0)
Cer [NS] (d18:2/24:2)
Cer [NS] (d18:2/25:1)
Cer [NS] (d18:2/26:1)
FA 19:1
FA 19:2
FA 22:2
FA 22:4
HexCer [NS] (d18:2/16:0)
lysoPC 20:1
lysoPC 22:5
PC(16:1/18:3)
PC(18:1/20:3)
PC(18:1/22:6)
PE(16:0/18:0)
PE(16:0/18:3)
PE(18:1/22:2)
PE(a16:1/18:0)
PE(a16:1/18:1)
PE(a18:1/20:0)
PE(a18:1/20:1)
PE(a18:1/22:2)
PG(16:0/22:6)
PG(22:5/22:6)
PI(16:0/18:0)
PI(16:0/18:1)
PI(17:0/22:2)
PI(17:1/18:1)
PI(18:0/18:0)
PI(18:0/18:3)
PI(18:1/20:1)
PI(18:1/20:2)
PI(18:1/22:2)
PS(16:0/20:4)
PS(17:1/18:1)
PS(18:0/20:5)
PS(18:1/18:3)
PS(18:1/22:1)
PS(18:1/22:6)
PS(22:0/18:1)
SM(d18:0/16:0)
SM(d18:1/16:0)
SM(d18:1/22:1)
HexCer [NS] (d18:1/14:0)
FA 20:6
PC(14:0/14:1)
PC(14:0/15:0)
PC(14:0/16:1)
PC(14:1/16:0)
PC(14:1/16:1)
PC(15:0/16:1)
PC(16:0/17:0)
PC(16:1/16:2)
PC(16:1/18:2)
PC(16:1/20:3)
PC(17:0/20:3)
PC(18:0/20:5)
PC(18:1/20:5)
PC(18:1/22:5)
PC(18:2/20:4)
PE(14:0/16:0)
PE(16:0/16:0)
PE(16:0/16:1)
PE(16:0/20:1)
PE(a16:1/18:3)
PE(a18:1/20:5)
PE(a18:1/22:5)
PE(a18:1/22:4)
PE(a18:1/22:5)
PE(18:0/18:1)
PE(16:1/18:2)
PE(17:0/16:1)
PI(17:0/20:3)
PI(18:0/20:5)
